# Ethanol consumption inhibits T_FH_ cell responses and the development of autoimmune arthritis

**DOI:** 10.1038/s41467-020-15855-z

**Published:** 2020-04-24

**Authors:** Vugar Azizov, Katharina Dietel, Franziska Steffen, Kerstin Dürholz, Julia Meidenbauer, Sébastien Lucas, Michael Frech, Yasunori Omata, Narges Tajik, Lisa Knipfer, Anne Kolenbrander, Silvia Seubert, Dennis Lapuente, Maria V. Sokolova, Jörg Hofmann, Matthias Tenbusch, Andreas Ramming, Ulrike Steffen, Falk Nimmerjahn, Ralf Linker, Stefan Wirtz, Martin Herrmann, Vladimir Temchura, Kerstin Sarter, Georg Schett, Mario M. Zaiss

**Affiliations:** 1 Department of Internal Medicine 3, Rheumatology and Immunology, Friedrich-Alexander-University Erlangen-Nürnberg (FAU) and Universitätsklinikum Erlangen, Erlangen, Germany; 2 Deutsches Zentrum für Immuntherapie (DZI), Erlangen, Germany; 3 Department of Internal Medicine 1, FAU Erlangen, Germany; 4 Department of Molecular and Medical Virology, Ruhr-University, Bochum, Germany; 5 Department of Neurology, FAU Erlangen, Germany; 6 Department of Neurology, University Hospital Regensburg, Regensburg, Germany; 7 Institute of Clinical and Molecular Virology, University Hospital Erlangen, FAU Erlangen, Germany; 8 Division of Biochemistry, Department of Biology, FAU Erlangen, Germany; 9 Department of Biology, Institute of Genetics, FAU Erlangen, Germany

**Keywords:** Immunology, Autoimmunity, Rheumatoid arthritis

## Abstract

Alcohol consumption is a consistent protective factor for the development of autoimmune diseases such as rheumatoid arthritis (RA). The underlying mechanism for this tolerance-inducing effect of alcohol, however, is unknown. Here we show that alcohol and its metabolite acetate alter the functional state of T follicular helper (T_FH_) cells in vitro and in vivo, thereby exerting immune regulatory and tolerance-inducing properties. Alcohol-exposed mice have reduced Bcl6 and PD-1 expression as well as IL-21 production by T_FH_ cells, preventing proper spatial organization of T_FH_ cells to form T_FH_:B cell conjugates in germinal centers. This effect is associated with impaired autoantibody formation, and mitigates experimental autoimmune arthritis. By contrast, T cell independent immune responses and passive models of arthritis are not affected by alcohol exposure. These data clarify the immune regulatory and tolerance-inducing effect of alcohol consumption.

## Abstract (web summary)

Moderate consumption of alcohol is associated with protection from some autoimmune diseases. Here the authors show that ethanol and its metabolite acetate can protect mice from collagen-induced arthritis and provide evidence that the mechanism of this effect might be via inhibition of the effector function of T follicular helper cells.

## Introduction

Genetic predisposition accounts for approximately thirty percent of the risk of autoimmune disease. The other 70% of the risk is due to environmental factors, such as toxic chemicals, dietary components, gut dysbiosis, and infections ultimately leading to loss of self-tolerance^1,2^. Ethanol (C_2_H_5_OH, EtOH; commonly referred to as alcohol) is a part of dietary components previously shown to affect the immune system. Alcohol has combined effects on innate and adaptive immunity, which can significantly weaken the host defense at higher doses, predisposing chronic drinkers to infections (reviewed elsewhere^3^). Furthermore, toxic effects of alcohol on the immune function are underscored by the observation that fetal alcohol exposure interferes with the development of a functional immune system^4^. Moderate alcohol consumption may thus have immune modulatory effects, which may inhibit the development of autoimmune diseases. Indeed, moderate alcohol consumption has consistently been identified as protective factor for the onset of rheumatoid arthritis (RA)^5,6^ and systemic lupus erythematosus (SLE)^7^.

The mechanism by which alcohol mitigates the development of autoimmune diseases, such as RA, is unknown. A general anti-inflammatory and anti-analgesic effect of alcohol that dampens symptoms in arthritis has been suspected. However, such a generalized anti-inflammatory effect does not sufficiently explain the observed lower incidence of disease onset of RA^5,6,8–10^ in humans and mice^11^. Experimental evidence on the mechanism of the immunomodulatory effect of alcohol is more than scarce to date. Alcohol is metabolized in the cells by two enzymes, alcohol dehydrogenase (ADH) and aldehyde dehydrogenase (ALDH)^12^. These enzymes help break apart the ethanol molecule, supporting its elimination from the body. ADH metabolizes alcohol to acetaldehyde, a highly toxic substance and known carcinogen. Hence, in a second step, acetaldehyde is rapidly metabolized to acetate^12^. Acetate is also secreted by the gut microbiota after fermentation of fiber-rich diets and has immunomodulatory properties^13–15^. Here, we show that acetate too, as the prominent metabolite of alcohol, potently modulates T follicular helper (T_FH_) cell function.

Alcohol and acetate have a wide variety of immunomodulatory effects. Here we present one of these effects, linking alcohol-exposure and acetate-exposure to mitigation of functional T_FH_ and B cell conjugate formation, B-cell activation, and subsequent decrease in autoantibody response leading to tolerance and inhibition of the onset of arthritis.

## Results

### Alcohol consumption inhibits arthritis onset

**Fig. 1:**
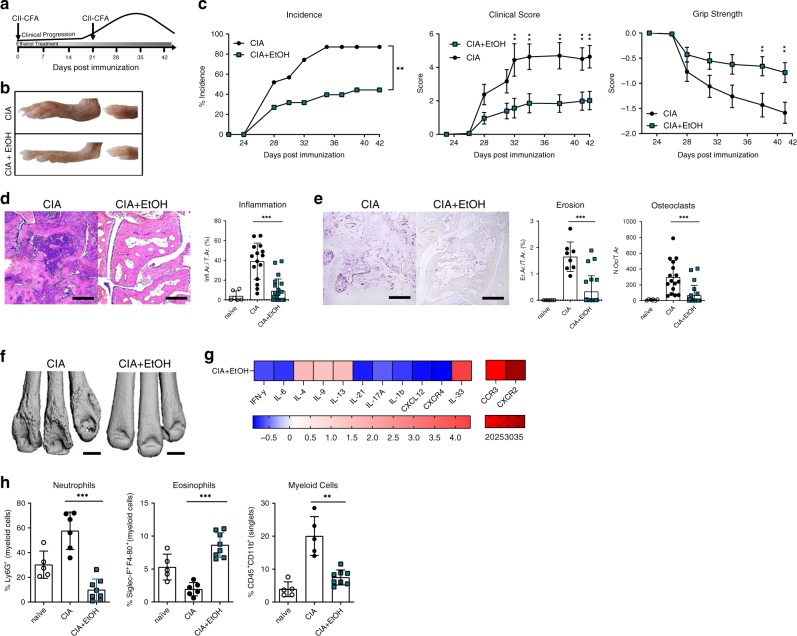
Alcohol exposure inhibits arthritis. **a** Schematic overview of the experimental setup in DBA/1 mice induced for collagen arthritis (CIA). **b** Representative images of hind paws and **c** incidence, clinical score and grip strength of vehicle-exposed mice (CIA, *n* = 10 mice) or alcohol-exposed mice (CIA + EtOH, *n* = 10 mice). **d** Representative images of H&E stained paw sections and histological quantification of inflammation area per tissue area (Infl. Ar./T.Ar.) in the hind paws (combined three independent experiments, naive *n* = 6, CIA, *n* = 8, CIA + EtOH, *n* = 21 mice). Scale bar, 500 µm. **e** Representative images of TRAP stained paw sections and histological quantification of eroded area per tissue area (E.Ar./T.Ar.) and osteoclast number per tissue area (N.Oc./T.Ar.) in the hind paws (combined three independent experiments, naive *n* = 6, CIA, *n* = 16, CIA + EtOH, *n* = 21 mice). Scale bar, 500 µm. **f** Representative µCT images of the tarsal joints of vehicle-exposed mice (CIA) or alcohol-exposed mice (CIA + EtOH). Scale bar, 200 µm. **g** Heat map exhibition of Log_2_ average fold change of relative mRNA expression levels of cytokine and chemokine mediators in the inflamed paws analyzed by RT-PCR at 42 dpi. **h** Flow-cytometry quantifications of neutrophils, eosinophils and monocytes at 42 dpi in cell suspensions from hind paws (naive *n* = 5, CIA, *n* = 6, CIA + EtOH, *n* = 8 mice). *N* number represents number of animals used per experiment. Data shown from one of three independent experiments and expressed as mean ± SD, except for **d** and **e**, which represent combined data. Statistical difference was determined by two-way ANOVA (**c**) or Students two-tailed *t*-test (**d**, **e**, **g**, **h**). *p < 0.05; **p < 0.01; ***p < 0.001.

To investigate the effects of alcohol on arthritis, we induced mice for collagen-induced arthritis (CIA) by immunizing DBA/1 mice with CII in CFA on days 0 and 21 and simultaneously started treatment with either 10% (vol/vol) ethanol in drinking water (CIA + EtOH) or water alone starting on day 0 (Fig. 1a). Consumption of ethanol was confirmed by measuring serum ethanol levels and by quantifying the amount consumed using individual metabolic cages (Supplementary Fig. 1a). At 42 days post immunization (dpi), hind and front paws of the ethanol-exposed group had strongly reduced symptoms of arthritis as shown by reduced swelling of the foot pad (Fig. 1b). Ethanol-exposed mice had 40% lower incidence of CIA, and a decrease of more than 50% in the severity of arthritis as compared to controls (Fig. 1c). Histological analysis of the hind paws showed significantly less synovial inflammation in ethanol-exposed CIA mice as compared to controls (Fig. 1d). In addition, we observed significantly lower bone erosion and osteoclast numbers in ethanol-exposed CIA mice (Fig. 1e). Micro-computed tomography (µCT) of the hind paws of ethanol-exposed CIA mice indicated no disease progression in comparison with the control group (Fig. 1f).

As demonstrated by gene expression analysis of whole-paw homogenates taken at 28 dpi, ethanol exposure reduced type 1 effector cytokine levels (IL-6, IL-1β, IFNγ) but increased IL-33 and cytokines associated with type 2 immune responses (IL-4, IL-9, IL-13) (Fig. 1g). IL-21 and IL17A, known key cytokines detected in RA joints, were significantly reduced (Fig. 1g). This observation is in accordance with previous data showing that type 2 immune responses attenuate clinical signs of arthritis^16,17^. Gene expression of Cxcl12 and CXCR4 and the numbers of neutrophils and monocytes were decreased (Fig. 1g, h), while Ccr3 and CXCR2 gene expression were increased in ethanol-exposed mice, correlating with flow cytometry analysis of increased eosinophil numbers in the joints (Fig. 1g, h). Ethanol consumption had no impact on T cell populations in spleens and lymph nodes of CIA mice (Supplementary Fig. 1b). Moreover, we could not observe any direct effect of ethanol on in vitro T cell differentiation assays into Th1, 2, 9 or 17 cells (Supplementary Fig. 1c) while T regulatory cells (Tregs) slightly decreased upon ethanol exposure (Supplementary Fig. 1c). Together, these data showed that ethanol exposure potently decreased the incidence and symptoms of an active T cell driven model of arthritis, accompanied by decreased pro-inflammatory effector cytokine production and immune effector cell influx into the joints while not directly affecting the Th cell subsets.

### Alcohol inhibits the initiation stage of autoimmunity

**Fig. 2:**
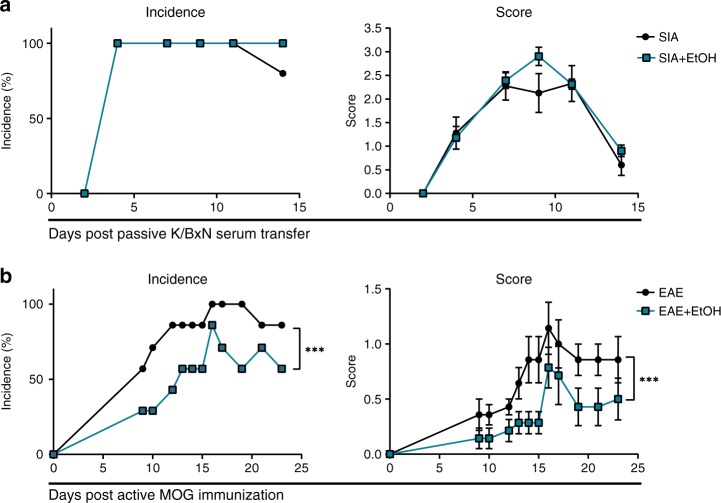
Alcohol inhibits active but not effector models of autoimmune inflammatory disease. **a** Incidence and clinical score of mice with K/BxN serum induced arthritis (SIA) exposed to vehicle (SIA *n* = 5 mice) or alcohol (SIA + EtOH, *n* = 10 mice). **b** Incidence and clinical score of experimental autoimmune encephalomyelitis (EAE) exposed to vehicle (EAE, *n* = 7 mice) or alcohol (EAE + EtOH, *n* = 7 mice). *N* number represents number of animals used per experiment. Data shown from one of three independent experiments and expressed as mean ± SD. Statistical difference was determined by two-way ANOVA. *p < 0.05; **p < 0.01; ***p < 0.001.

To address the question whether the observed attenuating effect of ethanol on arthritis incidence and severity is within the effector stage or the initiation stage of the immune response, we analyzed the effect of ethanol exposure in mice following passive K/BxN serum transfer. In this arthritis model the symptoms are induced by transferred autoantibodies, not requiring T cell responses and specific antibody generation. Neither the incidence, nor the clinical score of arthritis differed between ethanol-exposed and control mice following K/BxN serum transfer (Fig. 2a). Another disease model, experimental autoimmune encephalomyelitis (EAE), a model for multiple sclerosis, requires a T cell response and specific antibody generation akin to CIA. We observed attenuated clinical disease scores and lower incidence of EAE in ethanol-exposed mice compared to controls (Fig. 2b). Together, these experiments suggest that alcohol affects the early initiation stage of disease rather than the later effector stages.

### Alcohol exposure reduces IgG response

**Fig. 3:**
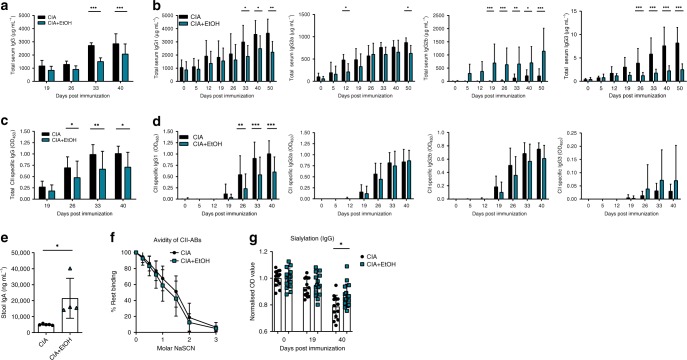
Alcohol exposure alters dynamics of IgG response. **a**, **b** Time course of the levels of total serum IgG (CIA, *n* = 7, CIA + EtOH, *n* = 5 mice) **a** and IgG subclasses **b** from mice induced for CIA and exposed to vehicle (CIA, *n* = 6 mice) or alcohol (CIA + EtOH, *n* = 6 mice). **c**, **d** Time course of the levels of collagen type 2 (CII)- specific serum IgG (CIA, *n* = 7, CIA + EtOH, *n* = 6 mice) **c** and IgG subclasses **d** from mice induced for CIA and exposed to vehicle (CIA, *n* = 6 mice) or alcohol (CIA + EtOH, *n* = 6 mice). **e** Stool IgA levels of mice induced for CIA and exposed to vehicle (CIA, *n* = 5 mice) or alcohol (CIA + EtOH, *n* = 5 mice). **f** Avidity of CII specific antibodies determined with NaSCN elution (CIA, *n* = 7 mice, CIA + EtOH, *n* = 5 mice). **g** Sialylation status of total serum IgG from mice induced for CIA and exposed to vehicle (CIA, *n* = 6 mice) or alcohol (CIA + EtOH, *n* = 6 mice). N number represents number of animals used per experiment. Data shown from one of three independent experiments and expressed as mean ± SD. Statistical difference was determined by two-way ANOVA (**a**, **b**, **c**, **d**, **f**, **g**) or Students two-tailed *t*-test (**e**). *p < 0.05; **p < 0.01; ***p < 0.001.

To further assess the role of ethanol in the initiation stage of CIA, we explored total IgG serum levels in mice induced for CIA. Ethanol-exposed mice exhibited lower total IgG levels starting from 19 dpi and reaching significance at 33 dpi compared to controls (Fig. 3a). Analysis of IgG subclasses showed reductions in levels of IgG1, IgG2a, IgG3, while IgG2b levels increased (Fig. 3b). CII-specific IgG was significantly decreased in ethanol-exposed mice starting from 26 dpi (Fig. 3c). A closer look at CII-specific IgG subclasses showed a decrease in IgG1, but unchanged IgG2a, IgG2b, or IgG3 levels (Fig. 3d). Based on the observed differences in antibody response, we advanced in exploring the avidity of CII-specific total IgG. As consumed ethanol is taken up and processed in the intestines, we also analyzed intestinal IgA production, and found increased levels of IgA in stool samples of ethanol-fed CIA mice (Fig. 3e) Interestingly, the avidity of CII-specific antibodies was not altered by ethanol consumption (Fig. 3f). We further checked CII-specific IgG sialylation and found significantly increased sialylation of IgG on 40 dpi in ethanol-exposed mice (Fig. 3g). This increase in sialylation of antibodies in ethanol-exposed CIA mice indicates a switch from a pro- to an anti-inflammatory state^18^. Altogether, these data show that upon ethanol exposure, IgG production is decreased and anti-inflammatory characteristics of IgG are enhanced, while affinity maturation does not seem to be affected.

### Alcohol increases Th1 and GC B cell number in steady state

Having observed a mitigating effect of ethanol on adaptive T-/B- cell mediated autoimmunity, we next tested if ethanol principally skews gut barrier function and adaptive immune cell function under steady state conditions. Gut leakiness did not change after a 3 week ethanol exposure in WT mice not induced for CIA (Supplementary Fig. 1d). Furthermore, most T cell lineages were unchanged after ethanol exposure with the exception of a moderate increase of splenic Th1 cells (Supplementary Fig. 1e). In addition, a moderate increase of GC B cells was observed (Supplementary Fig. 1f), while the numbers of T_FH_ cells, which play an essential role in the formation of GCs, as well as their doublets with B cells were not affected by ethanol (Supplementary Fig. 1g).

### Alcohol has inhibitory effect on B cell response

**Fig. 4:**
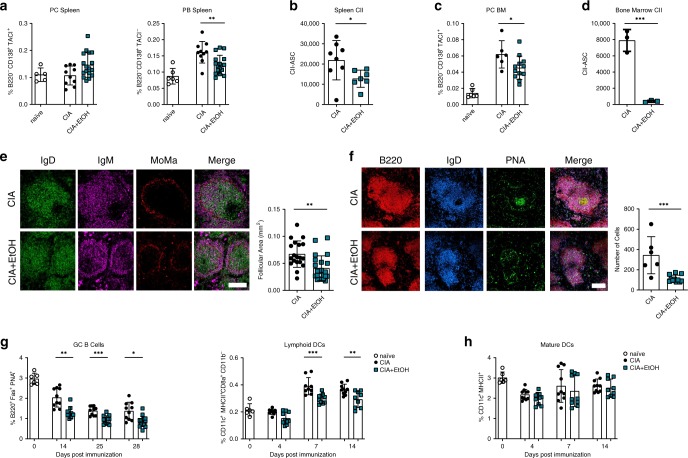
Alcohol exposure has pronounced effects on germinal center B cells. **a** Percentage of splenic plasma cells (B220^-^ CD138^+^ TACI^+^) and plasmablasts (B220^−^ CD138^+^ TACI^−^) of naïve mice (*n* = 6 mice) and mice induced for CIA and exposed to vehicle (CIA, *n* = 10 mice) or alcohol (CIA + EtOH, *n* = 17 mice). **b** total splenic CII specific cells determined via ELISpot (CIA, *n* = 8 mice, CIA + EtOH, *n* = 7 mice), **c** percentage of bone marrow plasma cells (B220^−^ CD138^+^ TACI^+^) (naive *n* = 6 mice, CIA, *n* = 6 mice, CIA + EtOH, *n* = 12 mice) and **d** total bone marrow CII specific cells determined via ELISpot in mice induced for CIA and exposed to vehicle (CIA, *n* = 3 mice) or alcohol (CIA + EtOH, *n* = 3 mice). **e** Representative immunofluorescence images of spleens from mice induced for CIA and exposed to vehicle (CIA, *n* = 7 mice) or alcohol (CIA + EtOH, *n* = 8 mice) at 28 dpi. Quantified by Image J area measure tool. Cryosections were stained for B cell follicle by IgG (green), IgM (magenta), and MoMa (red). Scale bar, 100 μm. **f** Representative immunofluorescence images of splenic cryosections for identification of germinal center B cells with B220 (red), IgD (blue), and PNA (green). Quantified by the average number of B220^+^ PNA^hi^ cells (CIA, *n* = 6, CIA + EtOH, *n* = 12 mice). Scale bar, 200 μm. **g** Flow-cytometry analysis of splenic germinal center B cells (B220^+^ Fas^+^ PNA^+^) and lymphoid dendritic cells (CD11c^+^ MHCII^+^ CD8a^+^ CD11b^−^) as well as **h** mature dendritic cells (CD11c^+^ MHCII^+^) of naive mice (*n* = 6 mice) and mice induced for CIA and exposed to vehicle (CIA, *n* = 10 mice) or alcohol (CIA + EtOH, *n* = 10 mice). *N* number represents number of animals used per experiment. Data shown from one of three independent experiments and expressed as mean ± SD. Statistical difference was determined by two-way ANOVA (**g**, **h**) Students two-tailed *t*-test (**a**, **b**, **c**, **d**, **e**, **f**). *p < 0.05; **p < 0.01; ***p < 0.001.

The observed reduction in IgG serum levels after ethanol consumption in arthritic mice prompted us to more closely analyze B cells in the spleen and the bone marrow of CIA mice with and without ethanol exposure (Supplementary Fig. 2a). Numbers of splenic plasma B cells (PC) were unchanged, but numbers of plasmablasts (PB) were decreased in ethanol-exposed CIA mice (Fig. 4a). More importantly, numbers of B cells producing CII-specific IgG were also decreased in ethanol-exposed CIA mice (Fig. 4b). In support of these findings, bone marrow plasma cells (PC BM), and CII-specific IgG producing B cells were significantly reduced upon ethanol exposure (Fig. 4c, d). To investigate, if B cell maturation or GC reaction were affected, cryosections of the spleens from ethanol-exposed and control CIA mice at 14 dpi were stained for B220, Peanut Agglutinin (PNA), IgD, IgM, and monocytes/macrophages (MoMa). Ethanol-exposed CIA mice exhibited less proliferation and activation of GC B cells than controls as shown by distinctly reduced follicular areas of defined B cell follicles (Fig. 4e) and decreased GC B cell numbers illustrated by lower B220/IgD/PNA expressing cells (Fig. 4f). Further flow cytometry analysis showed decreased splenic GC B cells from 14 dpi onward (Supplementary Fig. 2b) as well as significantly decreased numbers of lymphoid dendritic cells (DC) (Supplementary Fig. 2c) as early as 4 dpi in ethanol-exposed CIA mice (Fig. 4g) while mature DCs were not affected (Fig. 4h). Overall, the observed decrease in antibody titers (Fig. 3) can be attributed to a decrease in proliferation and activation of B cells in the GCs of CIA mice.

### Alcohol exposure suppresses IL-21 secretion by T_FH_ cells

**Fig. 5:**
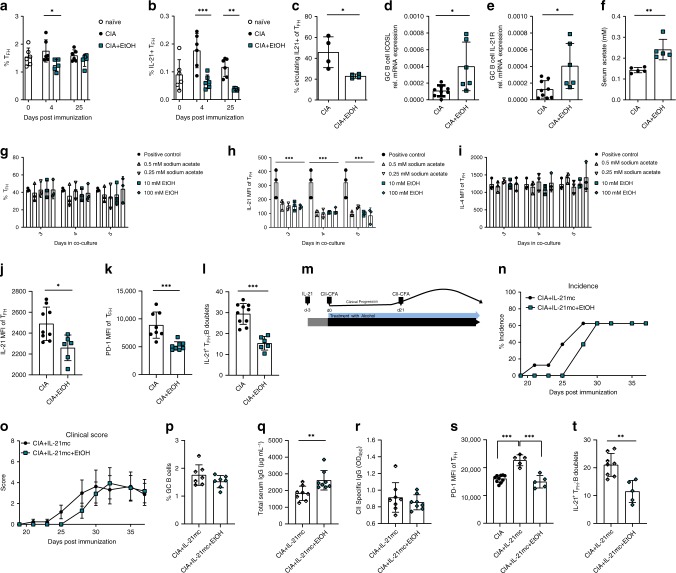
Alcohol exposure suppresses T_FH_ cell response. Flow cytometric analysis of splenic **a** T_FH_ and **b** IL-21 producing T_FH_ cells in naïve mice, CIA mice exposed to vehicle (CIA) or alcohol (CIA + EtOH) (*n* = 6 mice per group) **c** Percentage of IL-21 producing circulating T_FH_ cells at 28 dpi in peripheral blood of the two treatment groups (*n* = 4 mice per group). **d** ICOSL and **e** IL-21R gene expression in sorted GC B cells in the two treatment groups (CIA, *n* = 10 mice, CIA + EtOH, *n* = 6 mice). **f** Serum acetate concentrations at 42 dpi (*n* = 5 mice per group). **g** Percentage of T_FH_ cells, **h** IL-21 MFI and **i** IL-4 MFI of T_FH_ cells in in vitro differentiation (**g**–**i** three independent experiments, *n* = 1 per experiment, three technical repeats). **j** IL-21 MFI (CIA, *n* = 10 mice, CIA + EtOH, *n* = 6 mice) and **k** PD-1 MFI of T_FH_ cells in CIA and CIA + EtOH mice (*n* = 8 mice per group). **l** Flow cytometry analysis of IL-21 producing T_FH_:B cell conjugates in CIA (*n* = 10 mice) and CIA + EtOH mice (*n* = 6 mice) expressed as percent of all T_FH_:B conjugates. **m** Experimental design of IL-21 overexpression in CIA model. **n** Incidence and **o** clinical score of arthritis in CIA mice treated with IL-21 minicircle DNA without (CIA + IL-21mc) and with alcohol exposure (CIA + IL-21mc+EtOH) (*n* = 8 mice per group). **p** Flow cytometry analysis of GC B cells CIA + IL-21mcCIA+IL-21mc+EtOH mice (*n* = 7 mice per group). **q** Total serum IgG and **r** CII-specific total serum IgG in CIA + IL-21mcCIA + IL-21mc+EtOH mice (*n* = 8 mice per group). **s** PD-1 MFI of T_FH_ cells in CIA mice (*n* = 10 mice), CIA + IL-21mc+EtOH mice (*n* = 5 mice), and CIA+IL-21mc mice (*n* = 5 mice). **t** Flow cytometry analysis of IL-21 producing T_FH_:B cell conjugates as percentage of all T_FH_:B conjugates (CIA + IL-21mc, *n* = 8 mice, CIA + IL-21mc + EtOH, *n* = 5 mice). *N* number represents number of animals used per experiment. Data shown from one of three independent experiments and expressed as mean ± SD. Statistical difference was determined by one-way (**s**), two-way ANOVA (**g**, **h**, **i**, **n**, **o**) or Students two-tailed *t*-test (**a**–**f**, **j**–**l**, **p**–**r**, **t**). *p < 0.05; **p < 0.01; ***p < 0.001.

Next, we checked the effect of ethanol on T_FH_ cells, which are vital for induction of antibody production by B cells at the T-B interaction sites in the GC^19^. T_FH_ cell numbers were decreased already at 4 dpi in ethanol-exposed CIA mice, however, this difference faded following the 2^nd^ CII challenge at 21 dpi (Fig. 5a). IL-21 is a major cytokine produced by T_FH_ cells and was shown to play a key role in B cell antibody response as well as antibody class switch^20^. Flow cytometry analysis (Supplementary Fig. 3a) revealed defects in IL-21 secretion of T_FH_ cells of ethanol-exposed CIA mice as opposed to controls (Fig. 5b). We confirmed IL-21 antibody specificity by transfecting HepG2 cells with IL-21mc followed by intracellular staining (Supplementary Fig. 3b). Significantly decreased numbers of IL-21^+^ T_FH_ cells were observed as early as 4 dpi in ethanol-exposed mice, and this difference continued to increase throughout the course of the experiment (Fig. 5b). Of note, circulating IL-21^+^ T_FH_ cells started to decline at later time point of 28 dpi during the CIA clinical time course (Fig. 5c). Due to the intimate relationship between GC B cells and T_FH_ cells, we checked for ICOSL and IL-21R expression in sorted GC B cells. Interestingly, reduced production of IL-21 by T_FH_ cells was accompanied by a significant upregulation of ICOSL and IL-21R in the GC B cells of ethanol-exposed CIA mice (Fig. 5d, e). Given the importance of IL-21 within the direct T_FH_:B cell to cell interaction, we generated T_FH_ cells in vitro under most possible physiological conditions^21^ to confirm specifically the effects of ethanol on reducing IL-21 production by T_FH_ cells. Ethanol is ultimately metabolized into its immunologically active metabolite acetate^13^. We observed increased serum acetate levels in ethanol-exposed mice at 42 dpi during CIA, and this increase was detected as early as 5 days following ethanol-feeding (Fig. 5f and Supplementary Fig. 3c). Therefore, we also employed acetate in addition to the ethanol treatment in the following in vitro experiments. In line with our in vivo results, neither ethanol nor acetate treatment of in vitro differentiated T_FH_ cells^21^ changed total T_FH_ cell numbers (Fig. 5g and Supplementary Fig. 3d). Despite unchanged total T_FH_ cell numbers, we observed a decrease in overall cell numbers on days 4 and 5 post stimulation with 0.5 mM acetate and with 10 mM ethanol treatments (Supplementary Fig. 3e). Most importantly, IL-21^+^T_FH_ cells significantly decreased as shown by lower IL-21 mean fluorescence intensity (MFI) upon treatment with either ethanol or acetate (Fig. 5h). Interestingly, this was specific for IL-21 as IL-4 production was not affected in the same T_FH_ cells analyzed (Fig. 5i). Together, these in vitro experiments suggest that ethanol and its metabolite acetate specifically lower IL-21 production by T_FH_ cells.

This prompted us to further investigate the effect of ethanol on IL-21^+^T_FH_ cells in vivo in CIA exposed mice. Strikingly, we observed a reduction of the IL-21^+^T_FH_ cell population as well as a significant reduction in IL-21 MFI within this population in ethanol-exposed CIA mice. (Fig. 5j). Previously, a role of PD-1 in T_FH_ cell activation as well as T_FH_ cell concentration at the T-B cell border of the GCs has been reported^22^. Therefore, we proceeded by checking PD-1 expression levels of T_FH_ cells in ethanol-exposed CIA mice. Flow cytometry analysis of T_FH_ cells showed reduced PD-1 MFI in ethanol-exposed CIA mice (Fig. 5k). Consequently, as PD-1 is shown to be important for positioning of T_FH_ cells in the T-B cell border, we hypothesized that ethanol exposed mice exhibit less T_FH_:B cell doublets. T_FH_:B cell doublets represent the necessary cell conjugates that promote adaptive immune responses, where T_FH_ cells, via IL-21, regulate the fate of B cell antibody production in the GC^23^. Of note, flow cytometry analysis (Supplementary Fig. 3f) revealed reduced IL-21^+^T_FH_:B cell doublets in ethanol-exposed CIA mice compared to control mice (Fig. 5l).

### IL-21 reverses arthritis-mitigating effect of alcohol

To test whether increased IL-21 can overcome the attenuating effects of alcohol-exposure on arthritis, we injected IL-21 expressing mini-circle (mc) DNA^24^ 3 days before CII immunization (Fig. 5m). IL-21 serum levels were increased after IL-21mc injection as confirmed by ELISA (Supplementary Fig. 3g). Arthritis incidence and clinical scores in ethanol-exposed CIA + IL-21mc mice did not differ from non-exposed controls at 28 dpi although some delay in the course of arthritis was observed (Fig. 5n, o). Hence, IL-21 restored arthritis even in the presence of ethanol. In accordance, GC B cell populations were not different between arthritic IL-21mc/ethanol-exposed and IL-21mc/non-exposed mice (Fig. 5p). Total IgG levels were even higher in IL-21mc/ethanol-exposed mice (Fig. 5q), while no difference in CII-specific IgG levels was found (Fig. 5r).

While high systemic IL-21 levels can partially overcome the regulatory effect of ethanol in arthritis, IL-21 does not only affect T_FH_ cells. Down-regulation of NK cell activation markers CD49 and CD16 together with increased activation of CD8 T cells was observed after IL-21mc treatment (Supplementary Fig. 3h, i), a pattern that has previously been shown to associate with accelerated CIA^25,26^. Also, Th17 cells, one of the key driving Th cell type in arthritis^27^ were increased after IL-21mc treatment (Supplementary Fig. 3j). In accordance, IL-21mc treatment generally exacerbated CIA (Supplementary Fig. 3k). Thus, the rescue of the suppressive effects of ethanol on arthritis by IL-21 seem to be downstream of T_FH_ cells. This is supported that the effect of ethanol on PD-1 expression by T_FH_ cells (Fig. 5s) and the formation of IL-21^+^T_FH_:B cell doublets (Fig. 5t) was still decreased despite the presence of IL-21.

### Alcohol attenuates T cell driven immune responses

**Fig. 6:**
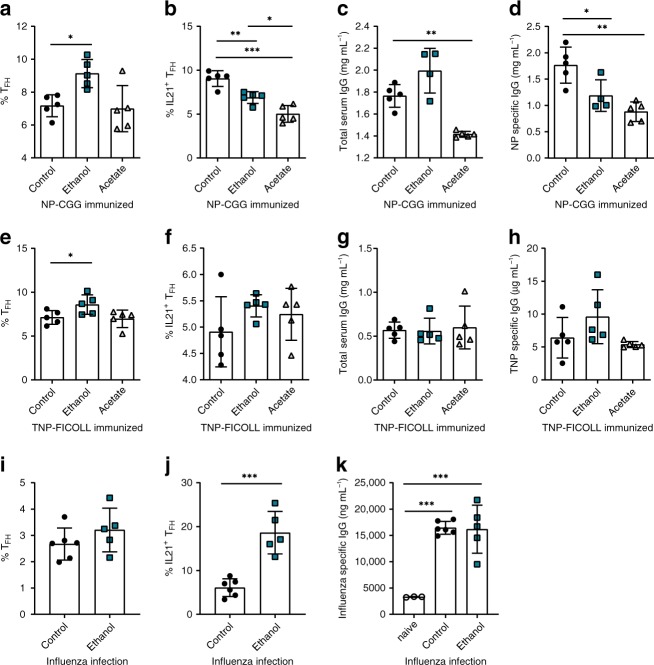
Alcohol exposure affects T cell driven immune responses. **a** Total T_FH_ (CD4^+^ B220^−^ PD-1^+^ CXCR5^+^ Bcl6^+^) cell numbers and **b** IL-21 producing T_FH_ (CD4^+^ B220^−^ PD1^+^ CXCR5^+^ Bcl6^+^ IL-21^+^) cell numbers in spleens of NP-CGG immunized mice treated either with water (control) or ethanol or acetate. **c** Total serum IgG and **d** NP-specific IgG levels in C57BL/6 mice immunized with NP-CGG (21 dpi). **e** Total T_FH_ (CD4^+^ B220^−^ PD-1^+^ CXCR5^+^ Bcl6^+^) cell numbers and **f** IL-21 producing T_FH_ (CD4^+^ B220^−^ PD1^+^ CXCR5^+^ Bcl6^+^ IL-21^+^) cell numbers in spleens of TNP-FICOLL immunized mice treated either with water (control) or ethanol or acetate. **g** Total serum IgG and **h** TNP-specific IgG levels in C57BL/6 mice immunized with TNP-FICOLL (21 dpi). **i** Total T_FH_ (CD4^+^ B220^−^ PD-1^+^ CXCR5^+^ Bcl6^+^) cell numbers and **j** IL-21 producing T_FH_ (CD4^+^ B220^−^ PD1^+^ CXCR5^+^ Bcl6^+^ IL-21^+^) cell numbers in spleens of influenza infected mice treated either with water (control) or ethanol. **k** Total serum influenza specific IgG levels in C57BL/6 mice (14 dpi). **a**–**h** *n* = 5 mice per group per experiment and representative experiment of three independent experiments shown. **i**–**k** *n* = 6 mice per group per experiment and representative experiment of two independent experiments shown. Data are expressed as mean ± SD. Statistical difference was determined by one-way ANOVA (**a**–**h**, **k**) or Students two-tailed *t*-test (**i**, **j**). *p < 0.05; **p < 0.01; ***p < 0.001.

To determine whether ethanol and its metabolite acetate generally affect T_FH_ dependent antibody responses, we analyzed C57BL/6J (WT) mice immunized with NP-CGG by flow cytometry (Supplementary Fig. 4a), an antigen that follows a T cell dependent pathway of immune response. While the T_FH_ cell numbers were not affected by acetate but increased by ethanol consumption (Fig. 6a), the frequencies and the absolute numbers of IL-21-producing T_FH_ cells following NP-CGG immunization in WT mice decreased after both treatments (Fig. 6b and Supplementary Fig. 4b). Acetate was more effective in decreasing total serum IgG levels (Fig. 6c). NP-CGG specific IgG titers were decreased in both alcohol- and acetate-exposed mice (Fig. 6d), further confirming our results observed in CIA. In comparison, when WT mice were immunized with TNP-FICOLL, an antigen that does not trigger T cell dependent immune response, a moderate increase in T_FH_ cell numbers was observed after ethanol exposure, but not after acetate (Fig. 6e). IL-21^+^T_FH_ cell numbers, however, remained unchanged by alcohol and acetate (Fig. 6f) and also serum IgG or TNP-FICOLL-specific IgG levels remained unchanged (Fig. 6g, h) overall supporting the data obtained in passive model of arthritis. We additionally checked the effects of alcohol on a viral infection model. It has been described for instance that high-level chronic alcohol consumption increases the severity of influenza^28^. However, moderate ethanol exposure in our setting did not dampen T_FH_ cells in the context of influenza infection and did not elevate the severity of the disease progression (Supplementary Fig. 4c). Thus, total T_FH_ cells and influenza IgG were not altered (Fig. 6i) and IL-21 producing T_FH_ cells were even increased. (Fig. 6j, k). Overall, these data support the concept that the immune regulatory effect of moderate doses of ethanol observed in arthritis is not necessarily accompanied by a decrease in host defense.

### Alcohol and acetate decrease IL-21, Bcl6, and PD-1 expression

**Fig. 7:**
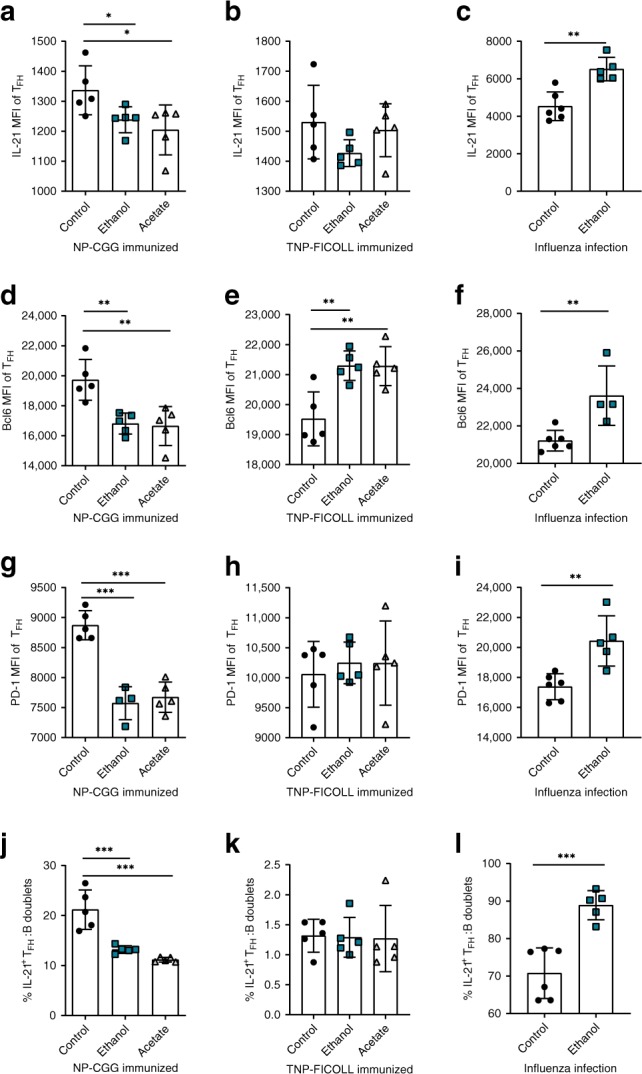
Alcohol and acetate decrease IL-21, Bcl6 and PD-1 expression. **a** Flow cytometry analysis of IL-21 MFI of T_FH_ cells in NP-CGG-, **b** TNP-FICOLL-immunized, **c** influenza infected mice treated with water (control), ethanol, or acetate. **d** Flow cytometry analysis of Bcl6 MFI of T_FH_ cells in NP-CGG-, **e** TNP-FICOLL-immunized, **f** influenza infected mice treated with water (control), ethanol, or acetate. **g** Flow cytometry analysis of PD-1 MFI of T_FH_ cells in NP-CGG-, **h** TNP-FICOLL-immunized, **i** influenza infected mice treated with water (control), ethanol, or acetate. **j** Flow cytometry analysis of IL-21 producing T_FH_:B conjugates as percentage of all T_FH_:B cell conjugates in NP-CGG-, **k** TNP-FICOLL-immunized, **l** influenza infected mice treated with water (control), ethanol, or acetate. **a**, **b**, **d**, **e**, **g**, **h**, **j**, **k** *n* = 5 mice per group per experiment and representative experiment of three independent experiments shown. **c**, **f**, **i**, **l** *n* = 6 mice per group per experiment and representative experiment of two independent experiments shown. Data are expressed as mean ± SD. Statistical difference was determined by one-way ANOVA (**a**, **b**, **d**, **e**, **g**, **h**, **j**, **k**) or Students two-tailed *t*-test (**c**, **f**, **i**, **l**). *p < 0.05; **p < 0.01; ***p < 0.001.

Based on reduced IL-21^+^ T_FH_ cell numbers and antibody titers in T-cell dependent immune responses upon ethanol exposure, we next investigated the underlying mechanism and responsible cellular network. When analyzing the MFI of IL-21 in IL-21^+^T_FH_ cells, we identified decreased IL-21 levels in NP-CGG immunized mice following ethanol or acetate feeding (Fig. 7a). Strikingly, the TNP-FICOLL immunized mice showed no difference (Fig. 7b) in IL-21 MFI between ethanol- and water-exposed mice and during influenza infection we observed even increased IL-21 MFI levels upon ethanol exposure (Fig. 7c). Recent studies suggested that IL-21 induces Bcl6, a master regulator of T_FH_ cells^29^. Interestingly and in agreement with the observed reduction of IL-21, Bcl6 levels were strongly reduced in T_FH_ cells upon ethanol-exposure of NP-CGG immunized mice (Fig. 7d). Contrary, T cell-independent TNP-FICOLL or influenza-immunized mice showed increased but not decreased Bcl6 levels following ethanol or acetate feeding (Fig. 7e, f). Based on recent literature showing that Bcl6 promotes PD-1 expression in T_FH_ cells^30,31^ we asked in which of our models PD-1 may be downregulated as well. Strikingly, only in the CIA and NP-CGG immunized mice, ethanol and acetate exposure reduced PD-1 expression (MFI level) (Figs. 5k and 7g). Following ethanol treatment PD-1 expression (MFI levels) were either unchanged in TNP-FICOLL or increased in influenza-immunized mice (Fig. 7h, i).

Recently, it was shown that PD-1 expression by T_FH_ cells is required for a successful positioning of T_FH_ cells in GCs in order to form a proper B cell conjugates (T_FH_:B cell doublets) and to promote antibody production^22^. Interestingly, following NP-CGG immunization IL-21^+^ T_FH_:B doublets decreased upon ethanol and acetate-exposure similar to CIA- induced ethanol-exposed mice (Figs. 7j and 5l). In TNP-FICOLL-immunized or influenza-infected mice, doublet formation remained unchanged or was even increased (Fig. 7k, l). Further analysis of T_FH_ cells within the T_FH_:B cell conjugates confirmed these observations. IL-21 expression (Supplementary Fig. 4d–f) and PD-1 expression (Supplementary Fig. 4g–i) within the T_FH_:B cell conjugates decreased only after NP-CGG immunization. In order to rule out the possibility that the observed changes in PD-1 levels within T_FH_:B cell doublets were due to PD-1 expression changes on B cells, we checked the B cell PD-1 MFI and found no differences (Supplementary Fig. 4j–l). These results strongly suggest that ethanol exposure prevents the onset of arthritis by causing a decrease in PD-1 expression of T_FH_ cells, inhibiting the secretion of IL-21 and the formation of functionally active T_FH_:B cell conjugates.

### Alcohol and acetate decrease number of T_FH_:B cell conjugates

**Fig. 8:**
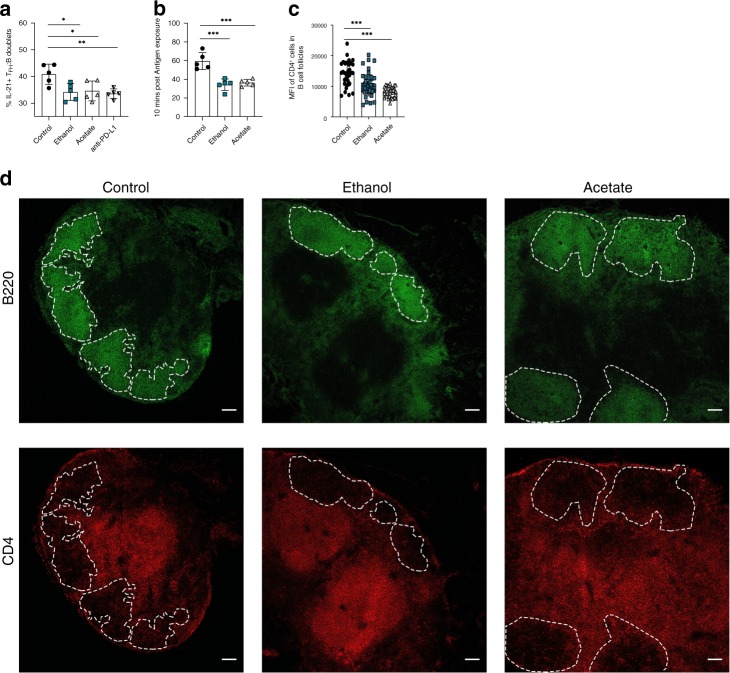
Alcohol and acetate prevent the formation of T_FH_:B cell conjugates. **a** Flow cytometry analysis of IL-21 producing T_FH_:B cell conjugates in co-cultures of T_FH_ and B cells activated with NP-CGG and treated either with water (control), or 10 mM ethanol, or 0.25 mM acetate, or 20 µg mL^−1^ anti-PD-L1 antibody. **b** Quantification of stable T_FH_:B cell conjugates formed in in vitro live cell imaging of T_FH_ and B cell co-cultures activated with NP-CGG and treated either with water (control), or 10 mM ethanol, or 0.25 mM acetate. **c** Immunofluorescence analysis of CD4^+^ T cells’ migration into B cell follicles in the draining lymph nodes of CIA mice exposed to either ethanol, acetate or vehicle. **d** Images representing CD4^+^ T cells within B cell follicles stained with anti-B220 (green) and anti-CD4 (red), followed by quantification of CD4 MFI within the B cell follicles. Scale bar, 100 µm. Raw images at 10.6084/m9.figshare.11793798. **a**, **b** *n* = 5 mice per group per experiment and representative experiment of three independent experiments shown. **c**, **d** *n* = 5 mice per group per experiment (control: 32, ethanol: 50, acetate: 38 follicles analyzed) and representative experiment of three independent experiments shown. Data are expressed as mean ± SD. Statistical difference was determined by one-way ANOVA. *p < 0.05; **p < 0.01; ***p < 0.001.

To confirm the importance of ethanol for T_FH_ cell mediated decrease in autoantibodies and subsequent decrease of clinical arthritis scores in vivo, we co-cultured sorted T_FH_ cells with B cells from C57BL/6J mice immunized with 100 µg of NP-CGG in Imject Alum 7 days prior. Later, NP-CGG was used to activate cells in vitro in the presence of ethanol or acetate. Anti-PD-L1 antibody was used as internal reference as it was reported previously^32^ to prevent functional IL-21^+^T_FH_:B cell conjugates. As hypothesized, flow cytometry analysis (Supplementary Fig. 4m) revealed that both ethanol and acetate showed similar efficacy in preventing the formation of IL-21^+^T_FH_:B cell conjugates in vitro, as with anti-PD-L1 treatment (Fig. 8a). Finally, we further confirmed our in vitro FACS approach by live cell, time-lapse microscopy. Therefore, we quantified total number of stable T_FH_:B cell interactions occurring under the effects of ethanol and acetate as compared to untreated control settings. Confirming our previous findings, strongly reduced T_FH_:B cell interactions were found in the presence of ethanol or acetate (Fig. 8b). As it was shown previously, PD-1 is required for proper positioning of T_FH_ cells in T/B cell border^22^. Therefore, we analyzed the migration of T_FH_ cells to T/B cell border by immune histology of the draining inguinal lymph nodes of ethanol and acetate-exposed CIA mice^33^. Interestingly, we found that there are less T_FH_ cells migrating into B-cell follicles upon ethanol or acetate exposure explaining the impaired T_FH_:B cell conjugate formation (Fig. 8c, d).

Altogether, in vitro observations show that ethanol and acetate have strong effects on T_FH_:B cell interplay, just as it is observed in vivo.

## Discussion

Ethanol is the principal psychoactive constituent in alcoholic beverages. Heavy alcohol consumption is a frequent social and health problem due to central nervous system (CNS) and liver toxicity^34,35^. On the other hand, it is also known that moderate alcohol consumption has immune regulatory and anti-inflammatory effects^36,37^. In support of this notion, several epidemiological studies have shown that alcohol consumption has an inhibitory effect on the frequency and severity of RA^8–10,38,39^. Still, the underlying mechanism by which alcohol mitigates RA has only partly been investigated. Jonsson et al. reported that alcohol-exposed DBA1 mice showed reduced testosterone serum levels two weeks after clinical arthritic onset and cells isolated from alcohol-exposed NMRI mice secreted less pro-inflammatory cytokines (TNF, MIP-1α) and showed reduced migration towards an artificial chemotactic stimulus than did cells from non-alcohol-exposed control mice^11^. However, while such general and late anti-inflammatory effects might explain the attenuated clinical arthritis symptoms, they do not sufficiently explain the reported lower incidence of RA in humans consuming alcohol^8–10,38,39^ suggesting more specific and early tolerance inducing effects of alcohol. Our study shows that alcohol alters the activity of T_FH_ cells by down-regulating key activation nodes such as Bcl6, PD-1 and IL-21, thereby preventing the formation of functional T_FH_:B cell conjugates and ultimately leading to a decline in autoantibody production and lower incidence of arthritis.

Recently it has been shown that T_FH_ cells are implicated in the outcome of autoimmune arthritis. Intestinal T_FH_ cells in Peyer’s patches (PP), specifically stimulated by gut commensal segmented filamentous bacteria (SFB), promoted K/BxN autoimmune arthritis by elevating their egress into systemic sites boosting autoantibody responses^40^. T_FH_ cells are crucial for B cell differentiation in the germinal centers (GC) and the production and class switch of high-affinity antibodies^41,42^. The role of T_FH_ cells and their primary product IL-21^43^ is important for T_FH_ cell development and is required for B cell differentiation and antibody production^44,45^. Mice deficient in the receptor for IL-21 (IL-21R)—that is expressed on T_FH_ and B cells -have significantly lower IgG1, IgG2b/2a and IgM titers than wild-type mice following immunization^23^. Conversely, high expression levels of IL-21 correlate with the T_FH_ cells’ enhanced capacity to facilitate antibody production by B cells^19^. It is important to note that IL-21 does not act on T_FH_ cells only but also boosts the cytotoxicity of CD8^+^ T cells and NK cells^42^ as well as induces Th17 cell differentiation^46^ which triggers antibody production and class switch recombination^47^.

**Fig. 9:**
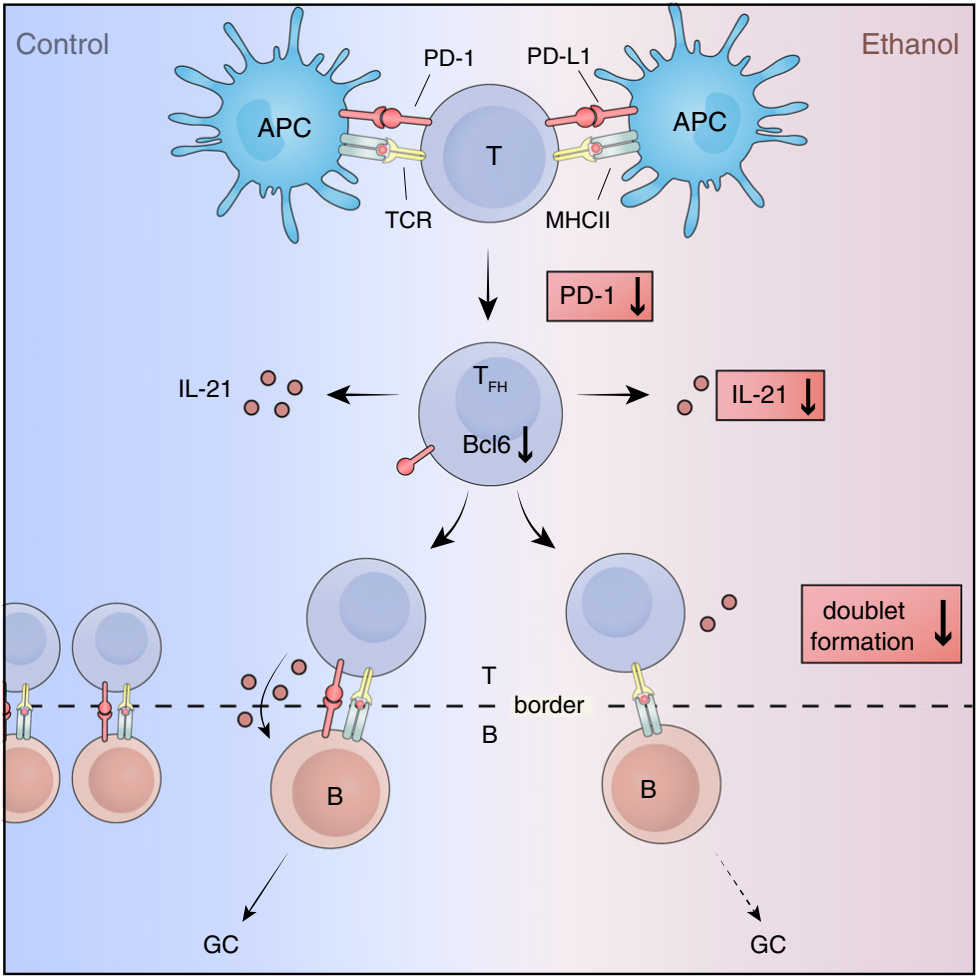
Effect of alcohol on immune response. Our findings indicate that the exposure to alcohol causes a reduction in T follicular helper cell master transcription factor Bcl6. Additionally, PD-1 and IL-21 are also reduced. Based on flow cytometry and immunohistology analysis, there are less T_FH_:B cell co-localization and conjugate formation, followed by observed lower autoantibody titers and mitigated disease progression.

To provide appropriate help to B cells, T_FH_:B cell conjugates form in the GC of secondary lymphoid organs with precise spatial and temporal coordination to yield antibody responses. PD-1 which is generally considered to be inhibitory on immune responses was found to be highly expressed on T_FH_ cells inside the GC^48,49^. Despite high PD-1 expression levels, T_FH_ cells are functional and sensitive to antigens presented by cognate B cells suggesting a different function of PD-1 in T_FH_ cells^50^. Recently, Shi et al. showed that PD-1 expression by T_FH_ cells is essential for their optimal spatial localization to GCs to form T_FH_:B cells conjugates primed for IL-21 production^22^. This coincides with our finding of reduced PD-1 expression on GC T_FH_ cells along with lower frequencies of functional IL-21^+^T_FH_:B cell conjugates as well as lower infiltration of B-cell follicles by T_FH_ cells in alcohol- and acetate-exposed CIA mice. We have graphically summarized the events we believe are affected by alcohol during an early immune response (Fig. 9). Similarly in vitro exposure to ethanol or acetate specifically reduced IL-21 secretion by T_FH_ cells and prevented formation of T_FH_:B cell conjugates to the same extent as did pharmacological blocking of PD-L1 on B cells. Bcl6 and PD-1 expression and IL-21 secretion by T_FH_ cells^51,52^ as well as GC reactions^29^ were also downregulated by alcohol in T cell dependent immunization models.

It is well known that autoimmunity precedes the onset of the inflammatory phase of RA^53^ and that titers and sialylation status of autoantibodies change before the onset of the disease^54,55^. Presence of autoantibodies against citrullinated proteins provides clear evidence for a central role of T- and B cell-driven autoimmunity in RA^38,56^. Several lines of evidence point to aberrant T_FH_ responses contributing to the development of RA^57–63^. Hence, T_FH_ cells are detected in the synovial tissue of patients with RA^63^. In addition, higher levels of IL-21 mRNA expression in peripheral blood mononuclear cells (PBMCs) and of IL-21 protein in the serum positively correlate with the disease severity and the levels of autoantibodies in RA patients^59,63^. Finally, although the expression of inhibitory receptors (such as CD200) can be important for the role of T_FH_ cells^62^, an increased frequency of CD4^+^CXCR5^+^ICOS^+^ T_FH_ cells and a higher T_FH_/Th_1_ ratio has been observed in RA^61,64^.

Systemic IL-21 neutralization in arthritis showed only modest, although significant, effects. Joint swelling and histological scores in IL-21R^−/−^ mice induced for CIA were attenuated comparable to the ones in IL-6^−/−^ mice, however single treatment with sIL21R.Fc protein did not show differences compared to the combined sIL21R.Fc/IL-6R treatment^65^. Another group reported attenuated clinical arthritis scores and lower total serum IgG1 levels in CIA DBA1 mice and in Lewis rats induced for antigen induced arthritis (AIA) following IL-21R.Fc treatment^66^. It has to be taken into account that IL-21R.Fc treatment was used in a therapeutic setting. Our data suggest that the primary mechanism of alcohol-meditated reduction in the incidence of arthritis lies in early cellular events, before the onset of arthritis^65,66^. Hence, inhibition of IL-21 may be better suited in preventing the transition from autoimmunity to inflammation rather than inhibiting the effector phase of the disease. Moreover, it has been shown that defective T_FH_ cell function caused by a reduction in PD-1 resulted in decreased T-cell dependent antibody responses independent of IL-21^67^. Our study also showed that PD-1 levels are consistently down-regulated on T_FH_ cells following EtOH treatment, suggesting that alcohol-feeding resembles a more complex physiology extending beyond the effects of IL-21, presumably also preventing functional T_FH_:B cell interaction. In line with this notion, IL-21 is not strictly confined to T_FH_ cells as it has been shown that IL-21 can also be expressed by CXCR5- negative T cells^68^. Consequently, one should not ignore the direct or indirect effects of ethanol and acetate on lymphocyte’s metabolism, migration speed, synapse formation and adhesion ability that could contribute to our findings. Unraveling such mechanisms would be of great benefit for further better understanding of the effects of nutrition on immunity. These data support the need for further studies investigating the potential to modulate the Bcl6/PD-1/IL-21 axis specifically in T_FH_ cells during the initiation phase of RA.

In summary, our data clarify the nature of the robust preventive and disease ameliorating effects of alcohol reported in epidemiological RA studies.

## Methods

### Mice

Eight-week-old female C57BL/6 mice were purchased from Charles River (Germany). Six- week-old female DBA/1 mice were purchased from Janvier (Germany). Mice were co-housed for 2 weeks prior to start of experiments. OT 2 mice were kindly provided by Prof. Diana Dudziak from Dermatology Department FAU Erlangen-Nuremberg. b12HL mice were kindly provided by Prof. David Nemazee from the Scripps Research Institute, La Jolla, CA, USA. Kaede mice were kindly provided by M. Tomura from the RIKEN Institute, Tokyo, Japan. All mice were housed and experiments were conducted under specific pathogen-free conditions. The animals receive water and feed ad libitum. The keeping rooms have a temperature of 22–23 °C and a humidity of 50–60%. There is also a 12-h light-dark rhythm in the holding rooms. Animals are kept in type II long cages, with max. five animals. All of the protocols for animal experiments were approved by the local ethics authorities of the Regierung von Unterfranken.

### Collagen-induced arthritis

CIA was induced in 8-week-old female C57BL/6J or DBA/1J mice by s.c. at the base of the tail with 100 μl with 0.25 mg chicken type II collagen (CII; Chondrex, Redmond, WA) in complete Freund adjuvant (CFA; Difco Laboratory, Detroit, MI), containing 5 mg mL^−1^ killed Mycobacterium tuberculosis (H37Ra). Mice were re-challenged after 21 days intradermal immunization in the base of the tail with this emulsion. The paws were evaluated for joint swelling and grip strength three times per week. Each paw was individually scored using a 4-point scale: 0, normal paw; 1, minimal swelling or redness; 2, redness and swelling involving the entire forepaw; 3, redness and swelling involving the entire limp; 4, joint deformity or ankylosis or both. In addition, grip strength of each paw was analyzed on a wire 3 mm in diameter, using a score from 0 to −4 (0, normal grip strength; −1, mildly reduced grip strength; −2, moderately reduced grip strength; −3, severely reduced grip strength; −4, no grip strength at all).

### Histology

Paw bones were fixed in 4% formalin for 24 h and decalcified in EDTA (Sigma-Aldrich). Serial paraffin sections (2 μm) were stained for H&E and tartrate resistant acid phosphatase (TRAP) using a Leukocyte Acid Phosphatase Kit (Sigma) according to the manufacturer’s instructions. Osteoclast numbers were quantified using a microscope (Carl Zeiss) equipped with a digital camera and an image analysis system for performing histomorphometry (Osteomeasure; OsteoMetrics).

### Micro-computed tomography

µCT imaging was performed using the cone-beam Desktop Micro Computer Tomograph µCT 40 by SCANCO Medical AG, Bruettisellen, Switzerland. The settings were optimized for calcified tissue visualization at 55 kVp with a current of 145 µA and 200 ms integration time for 500 projections/180°. For the segmentation of 3D-Volumes an isotropic voxel size of 8.4 µm and an evaluation script with adjusted greyscale thresholds of the operating system Open VMS by SCANCO Medical was used.

### Real-time PCR

Tissues were stored in RnaLater (Ambion) or directly transferred to TRIzol (Invitrogen). RNA was extracted according to the manufacturer’s instructions. Gene expression results are expressed as arbitrary units relative to expression of the house keeping gene β-Actin. Primer sequences are provided in Supplementary Table 1.

### Flow cytometry

Spleens were smashed and filtered through 40 μm gauze (BD Biosciences). Single-cell suspensions were then stained for flow cytometry with the following antibodies: CD4 Pacific blue (clone GK1.5), CD25 PE-cy7 (clone PC61), FoxP3 AlexaFluor (clone FJK-16a, eBioscience) were used in combination with a FoxP3 staining kit (eBioscience). T_FH_ cells were detected with the following antibodies: CD4 (clone RM4-5), B220 (clone RA3-6B2), CXCR5 (clone SPRCL5), PD-1 (clone 29 F.1A12), ICOS (clone 7E.17G9), Bcl6 (clone K112-91), and IL-21 (clone FFA21). Further antibodies: CD3 (clone 145-2C11), CD8α (clone 53-6.7), CD11b (clone M1/70), CD11c (clone N418), CD45 (clone 30-F11), CD138 (clone 281-2), F4/80 (clone BM8), Fas (clone 15A7), Ly6c (clone HK1.4), Ly6G (clone 1A8), MHCII (clone M5/114.15.2), TACI (clone ebio8F10-3), and Siglec F (clone ES22-10D8), PNA-biotin (Vector), Streptavidin-PE/CF594 (BD), NKp46 (clone 29A1.4), CD49 (clone DX5), CD16 (clone 93), IL-4 (clone 11B11), IL-17 (clone TC11-18H10.1), IL-9 (clone RM9A4). T_FH_:B cell doublet gating strategy is adopted from previously published article^69^ and is shown in Supplementary Fig. 3f.

### K/BxN serum induced model

K/BxN serum induced arthritis was induced by intraperitoneal (i.p.) injection of 200 μL pooled K/BxN serum. The swelling of fore and hind paws were measured three times per week. Development of arthritis was evaluated for each paw using a semi-quantitative scoring system (0–4 per paw; maximum score of 16).

### EAE and MOG immunization

For induction of EAE, male and female mice of 8–11-weeks age were anaesthetized (ketamine/xylazine 80 mg per kg/8 mg per kg) and received a total of 200 μg MOG35-55 and 200 μg CFA, containing 4 mg/ml M. tuberculosis (H37RA) administered by two subcutaneous injections of 50 μl emulsion left and right to the tail base. Pertussis toxin (200 ng per mouse) was applied intraperitoneally on days 0 and 2 p.i. The clinical evaluation was performed on a daily bases by a 5-point scale ranging from 0, normal; 1, limp tail, impaired righting; 2, gait ataxia; 3, paraparesis of hind limbs; 4, tetraparesis; 5, death. Mice were sacrificed if reaching a disease score of 4.

### Quantification of CII-specific antibody producing cells

For the detection of antibody-secreting cells from spleen and bone marrow, nitrocellulose microtiter plates were moistened first with 15 μL per well of 70% methanol for one minute. The plate was then washed three times with 200 μl per well PBS and coated with collagen (20 μg mL^−1^ in PBS) overnight at 4 °C. After washing six times with 150 μL per well of PBS, 150 μL per well of PBS/2% FCS was first blocked for 2 h at RT and then for 1 h at 37 °C. Subsequently, 1 × 10^6^ cells were incubated in 100 μL cell suspensions overnight at 37°C. After washing six times with 150 μL per well of PBS, the membrane of the plate was washed from below with H_2_O and detected with goat anti-mouse IgG-HRP (diluted 1:1000 in PBS/2% FCS) for 1 h at RT. After washing 10 times, 100 μL per well TMB substrate solution was added to the wells for about 10 min and quenched with 100 μL per well MQ-H_2_O. Subsequently, the membrane was washed four times with MQ-H_2_O and one time with H_2_O from below. After drying in the dark spots were counted by AID ELISpot Reader.

### Immunofluorescence

Spleens and draining lymph nodes (dLNs) were isolated, spleens were split to three pieces, tissues were embedded in Tissue-Tek® (Sakura, Staufen) and frozen at −80 °C. 8-µm thick slices were cut with Cryotom (Thermo Scientific). Slices were fixed with acetone, air dried, and frozen at −20 °C. For stainings, slides were thawed at RT for 15 min, rehydrated in PBS or PBS 1% BSA (dLNs) for 5 min, blocked for 30 min in IHC-Blocking buffer or PBS 1% BSA (dLNs) and then stained with the following antibodies: CD4-Alexa Fluor 647, B220-Alexa Fluor 488, IgM-Cy5 (Southern Biotech), IgD-Alexa Fluor 488, MoMa-Biotin (Southern Biotech), streptavidin-TRITC (Southern Biotech), and DAPI (Abcam) in IHC-staining buffer for 30 min at RT in dark humid chamber. Then, slides were washed twice for 10 min with 0.05 % Tween20 in PBS and one time with PBS only. Slides were then mounted with mounting medium (Abcam) and analyzed with Zeiss Axio Lab.A1 (Carl Zeiss) or Leica SP5 Confocal microscope. B cell follicles were identified by B220 + areas at ×100 total magnification and borders drawn by isolate/draw tool. Then, a channel was switched to CD4 signal, and CD4 MFI of T cells within the isolated region analyzed by Fiji^70^. In average about 3–6 non-sequential follicles were counted per animal. Blind analysis was applied to all image analysis.

### ELISA

Total serum IgG was detected by capturing mouse serum IgG by coating Nunc-Immuno Microwell 96-well plates (Sigma-Aldrich Cat# M0661-1CS) with 10 µg mL^−1^ of unconjugated goat anti-mouse (H + L) (Southern Biotech cat#1036-01) overnight at 4 °C. Wells were washed three times. All washing steps were performed with PBS + 0.05 %Tween20 (v/v). Wells were blocked with PBS + 3%BSA (w/v) for 2 h at RT. After washing, serum samples were serially diluted from 200x to 145800x in PBS + 1%BSA (w/v) and incubated with capture antibody for 2 h at RT. In parallel, unlabeled mouse-IgG (Southern Biotech cat#0107-01) was added as a defined standard to each plate. After washing, goat anti-mouse IgG-HRP (Southern Biotech cat#1030-05) was added at 5000x dilution in PBS + 0.05%Tween20 (v/v) for 1 h at RT. After wash, ELISA development solution (Thermo Scientific Cat# 34021) was used for developing and quantified on Magellan Tecan Microplate reader (Tecan Trading AG, Switzerland) at OD_450_ as well as OD_520_ as a reference wavelength.

CII, NP, and TNP-specific IgG ELISAs were performed by first coating Nunc-Maxisorp ELISA plates (Thermo Fischer cat#44-2404-21) with corresponding antigens: chicken type II collagen (CII; Chondrex, Redmond, WA), NP-BSA (LGC, Middlesex, UK), and TNP-BSA (LGC, Middlesex, UK) at 10 µg mL^−1^ concentration overnight at 4 °C. In CIA, IgA, IgG, and IgG subclasses were detected by antibodies from Bethyl. IgG and IgG subclasses from immunization experiments were detected by antibodies from Southern Biotec. Next day, wells were washed, blocked, serum dilutions added, and detected as described above.

### Calculation of CII-specific IgG avidity

The avidity was determined by^71^ coating microtiter plates with type II collagen at 10 µg mL^−1^ in order to select for CII specific IgGs. After mice sera were incubated, wells were washed three times with PBS-T followed by serially diluted NaSCN, washed, and quantified with standard ELISA detection method mentioned above. Percentage of bound CII specific IgG was calculated by the ratio of OD_450_ values of NaSCN exposed samples to NaSCN unexposed control wells.

### Identification of IgG sialylation

To identify differences in the sialylation of IgGs, microtiter plates were coated with 100 μL per well mouse IgG-F (ab′) 2 fragment antibody (diluted 1:100 in coating buffer) at 4 °C overnight. After washing with TBST, 200 μL per well gelatin buffer (warmed at 37 °C) was blocked on the shaker at 350 rpm for 1 h. After rinsing, one time for 10 min in the incubator at 37 °C, incubation was carried out with 100 μL per well of the diluted serum (1: 1000, saturated) for 1 h on the shaker. It was then washed twice with TBST and once with lectin buffer. The lectin SNA of Sambucus nigra binds to sialic acid, therefore it was detected with 100 μL per well biotinylated Elderberry Bark lectin (1:40,000, diluted in lectin buffer) for 1 h on the shaker. It was then washed three times and incubated with streptavidin-HRP (diluted 1:200 in TBST) for 30 min on the shaker. After washing again, 100 μL per well of TMB substrate solution was added to the wells for about 2 min and quenched with 100 μL per well of Stop Solution after color change of the solution. The OD was measured at λ = 450 nm and λ = 650 nm on the ELISA reader.

### SCFA measurements

Four to five replicates of frozen cecal samples (100 mg) or 50 µL of serum were weighed into a 2 ml polypropylene tube. The tubes were kept in a cool rack throughout the extraction. Thirty-three percent of HCl (50 µL for cecal contents or 5 µL for serum) was added and samples were vortexed for 1 min. One milliliter of diethyl ether was added, vortexed for 1 min and centrifuged for 3 min at 4 °C. The organic phase was transferred into a 2 ml gas chromatography (GC) vial. For the calibration curve, 100 μl of SCFA calibration standards (Sigma) were dissolved in water to concentrations of 0, 0.5, 1, 5, and 10 mM and then subjected to the same extraction procedure as the samples. For GCMS analysis 1 μl of the sample (4–5 replicates) was injected with a split ratio of 20:1 on a Famewax, 30 m ×  0.25 mm iD, 0.25 μm df capillary column (Restek, Bad Homburg). The GC-MS system consisted of GCMS QP2010Plus gas chromatograph/ mass spectrometer coupled with an AOC20S autosampler and an AOC20i auto injector (Shimadzu, Kyoto, Japan). Injection temperature was 240 °C with the interface set at 230 °C and the ion source at 200 °C. Helium was used as carrier gas with constant flow rate of 1 ml min^−1^. The column temperature program started with 40 °C and was ramped to 150 °C at a rate of 7 °C min^−1^ and then to 230 °C at a rate of 9 °C min^−1^, and finally held at 230 °C for 9 min. The total run time was 40 min. SCFA were identified based on the retention time of standard compounds and with the assistance of the NIST 08 mass spectral library. Full scan mass spectra were recorded in the 25–150 *m/z* range (0.5 s per scan). Quantification was done by integration of the extracted ion chromatogram peaks for the following ion species: *m/z* 45 for acetate eluted at 7.8 min, *m*/z 74 for propionate eluted at 9.6 min, and *m/z* 60 for butyrate eluted at 11.5 min. GCMS Solution software version 2.5 was used for data processing.

### In vitro differentiation of T cells

Naïve CD4 T cells were isolated from the spleens of C57BL/6 mice (Stemcell Technologies, Germany). Naïve CD4 T cells were cultured in R-10 medium supplemented with 0.5 µg mL^−1^ PMA, 1 µg mL^−1^ of Ionomycin, and Monensin (Biolegend, Germany) 96-well cell culture plates pre-coated with anti-CD3 antibody. For induction of differentiation into specific lineages, differentiation cocktails were added in the following manner, for Th1: 20 ng mL^−1^ of IL-12 p70 (Peprotech, Germany), 10 µg mL^−1^ aIL-4 (Peprotech, Germany), Th2: 10 µg mL^−1^ Anti-IFNγ (Invitrogen, Clone: XMG1.2), 100 ng mL^−1^ IL-4, Th9: 5 ng mL^−1^ rhTGFβ (Biolegend, cat# 580702), 10 µg mL^−1^ Anti-IFNγ, 10 ng mL^−1^ IL-4, Th17: 40 ng mL^−1^ IL-6 (Peprotech, Germany), 2 ng mL^−1^ rhTGFβ, Treg: 10 ng mL^−1^ IL-4.

### In vitro T_FH_ differentiation

For in-vitro differentiation of T_FH_ cells, purified (Miltenyi Biotec, Germany) dendritic cells from C57BL/6 mice, CD4 T cells from OT2 mice and B cells from b12HL cell mice were co-cultured for 6 days in the presence of HIV-derived virus‐like particles containing matched B- and T-cell epitopes (Env‐OT2‐VLPs) as follows^21^: 2 × 10^5^ T cells were plated in U-bottom 96 well plates in R10 medium. Dendritic cells (1:5, DC:T) from wild-type mice and B cells (1:2, B:T) from b12HL mice were co-cultured in the presence of 100 ng mL^−1^ of Env-OT2-VLPs. At days 3, 4, and 5 of co-culturing 10 mM and 100 mM of ethanol as well as 0.25 mM and 0.5 mM of acetate were added to the cells. For the intracellular staining against IL‐21 and IL‐4 2 μM monensin was added on day 6 and the cells were incubated for another 6 h before the analysis^21^.

### Adoptive transfer experiment

DBA/1J mice were hydrodynamically injected with 4 μg of IL-21 minicircle 3 days before CII-CFA immunization. Later collagen induced arthritis protocol was followed and clinical scores performed at the indicated time points.

### NP-CGG and TNP-FICOLL Immunizations

Female, 8-week-old C57BL/6 mice were purchased from Charles River (Germany). Starting one week before immunization, mice were given either 2% (w/v) Glucose water, 10% (v/v) Ethanol (Roche) and 2% (w/v) Glucose (Sigma), or 150 mM Acetate (Sigma), all feedings were changed every 3 days. For primary NP-CGG immunization, mice were then injected i.p. with 100 µg of NP-CGG (LGC, Middlesex, UK) in 200 µL of Imject Alum (Thermo Scientific) according to manufacturer’s instructions. Fourteen days later, mice were boosted with 100 µg of NP-CGG in 200 µL of alum. For TNP-FICOLL immunizations, mice were injected i.p. with 10 µg of TNP-FICOLL (LGC, Middlesex, UK) in 200 µL of Imject Alum (Thermo Scientific). For in vitro T_FH_ differentiation, B-cell receptor transgenic mice specific for HIV-1 Env protein (b12HL mice, in-house breeding) and T-cell receptor transgenic mice specific for chicken ovalbumin 323–339 in the context of I-A^b^ (OT2 mice, in‐house breeding) were used.

### Influenza infection model

Starting one week before infection, C57BL/6 mice were given either 2% (w/v) Glucose water or 10% (v/v) Ethanol (Roche) and 2% (w/v) Glucose (Sigma). All feedings were changed every 3 days and were continued throughout the infection. Mice were experimentally infected with 200 PFU of H1N1 A/Puerto Rico/8/1934 in 50 µL PBS. The inoculum was given intranasally under general anesthesia and weight loss was monitored daily. 14 days post infection, mice were sacrificed, bronchoalveolar lavages were performed in a total volume of 2 mL PBS, and spleen as well as lungs harvested.

### Total Influenza specific IgG ELISA

Ninety-six-well ELISA plates were coated with 5 × 10^5^ PFU heat-inactivated influenza particles (H1N1 A/Puerto Rico/8/1934) per well or polyclonal goat anti-mouse Ig (Southern Biotech, cat#5300-05) diluted in carbonate buffer overnight at 4 °C. Afterwards, free binding sites were blocked with 5% skimmed milk in PBS-T containing 0.05% Tween-20. After a washing step with PBS-T, diluted sera were added and incubated for 1 h. In parallel, specific amounts of mouse-Ig (Southern Biotech, cat#5300-01) were added as a defined standard to the anti-mouse Ig-coated wells. Subsequently, plates were washed and polyclonal anti-mouse IgG HRP-coupled secondary antibodies were added for 1 h (Dianova, cat#115-035-062). After a final wash and the addition of a homemade ECL substrate, the signals were detected with a microplate luminometer.

### Steady-state experiments

Female, 8-week-old C57BL/6 mice were fed 2% (w/v) glucose water, 10% ethanol (v/v) with 2% (w/v) glucose in water, or 150 mM sodium acetate in water for three weeks. At the conclusion of the experiment, mice were sacrificed and spleens collected for stimulation/blocking followed by flow cytometry analysis for Th_1_ (CD3^+^ CD4^+^ CD19^−^ IFNγ^+^), Th_2_ (CD3^+^ CD4^+^ CD19^−^ IL-4^+^), Th_17_ (CD3^+^ CD4^+^ CD19^−^ IL-17^+^), GC B cells (CD19^+^ B220^+^ Fas^+^ PNA^+^), and T_FH_ cells (CD4^+^ B220^−^ PD-1^+^ CXCR5^+^ Bcl6^+^). Gut leakiness was determined by feeding mice with either water, ethanol or acetate as mentioned above for two weeks followed by an intestinal permeability assay.

### Intestinal permeability assay

Mice fasting for 4 h were orally gavaged with 200 μL of FITC-dextran 4kD (440 mg kg^−1^ of body weight) (Sigma, Germany), and blood was collected 4 h later. FITC-dextran serum concentration was measured by flow cytometry at 490 nm excitation wavelength and detected at 530 nm emission filter. Standard curve of serially diluted FITC-dextran was used for data fitting.

### In vitro T_FH_ and B cell co-culture

C57BL/6 mice were intraperitoneally immunized with 100 µg of NP-CGG (LGC, Middlesex, UK) in 200 µL Imject Alum (Thermo Scientific) 7 days prior to sacrifice, at which point spleens were collected. Then, CD4^+^ CD19^−^ PD-1^+^ CXCR5^+^ T_FH_ and CD19^+^ B220^+^ B cells were sorted with Beckman Coulter MoFlo XDP cell sorter. Later, 10^5^ cells of each B and T_FH_ cells were co-cultured in R-10 medium and treated either with PBS, 10 mM ethanol, 0.5 mM acetate, or 20 µg mL^−1^ of PD-L1 neutralizing antibody (Biolegend, clone 10 F.9G2). NP-CGG was added to a final concentration of 20 µg mL^−1^ at the beginning of the experiment. 48 h later, cells were stained for FACS analysis as described before.

### Live cell microscopy

Kaede transgenic C57BL/6 mice were immunized with NP-CGG as described earlier. Spleens were harvested and single-cell suspension of these cells were prepared. Ninety percent of the spleenocytes were photoconverted by separately exposing cells to Spot UV curing equipment (Bluewave QX4, Dymax) at 405 nm wavelength and 14.9 W per cm^2^ (VisiCure, Dymax) for 150 s on ice. Once exposed to UV, cells were washed with FACS Buffer and filtered. T_FH_ cells were sorted as described earlier while B cells were isolated (Stemcell Technologies, Germany) using the rest 10% of the spleenocytes (not exposed to UV light). Two-well culture-inserts (Ibidi, Germany) were inserted into wells of 24-well cell culture plate. 10^5^ T_FH_ and B cells in R-10 medium were seeded into separate wells. Cells were allowed to settle for 6 h at 37 °C and 5% CO_2_. Later, the medium was removed, Two-well culture inserts removed, and wells gently refilled with either only R-10 medium or R-10 medium supplemented with 10 mM ethanol, or 0.25 mM acetate. The cells were then transferred to incubated and CO_2_ gassed Zeiss Cell Discoverer live imaging microscope. Before starting imaging, NP-CGG was added to a final concentration of 20 µg mL^−1^. Then images were taken by illumination at 470 nm and detection at 514/30 filter for green Kaede (unconverted Kaede B cells), illumination at 567 nm and detection at 592/25 for converted Kaede (T_FH_ cells) and brightfield setting with Apochromat ×5 objective in 2 minute intervals for a total of 1 h. Images were then analysed by a custom plugin for Fiji^70^. Positive cells were detected for each channel by applying a 3D Difference of Gaussian filter to the images and identifying local maxima. False positives were excluded by applying a manual threshold to the filtered images. Kaede and photoconverted Kaede, B cells and T_FH_ cells respectively, were counted as “in-contact” if the distance between the centers of the two cells was less than 10 µm.

### Statistical analysis

Data are expressed as mean ± SD unless otherwise indicated in the figure legends. All relevant data are available from the authors. Analysis was performed using a two-sided Student’s *t* test, single comparison or analysis of variance test for multiple comparisons (one-way or two-way ANOVA followed by Tukey’s or Bonferroni’s multiple comparisons test respectively). All experiments were conducted at least two times, unless otherwise indicated in the figure legends. *n*-numbers denote number of individual animals. *P*-values of 0.05 were considered significant and are shown as *p*0.05 (*), *p*0.01 (**), or *p*0.001 (***). Graph generation and statistical analyses were performed using Prism version 8 software (GraphPad, La Jolla, CA).

## Supplementary information

Supplementary Information

## Data Availability

The data that support the findings of this study are available from the corresponding author upon reasonable request. Raw images supporting Fig. 8 are deposited in Figshare 10.6084/m9.figshare.11793798. Raw data for the figures are available as a Source Data file.

## Source data

Source Data
