# Supplementary material for: Ethanol consumption inhibits TFH cell responses and the development of autoimmune arthritis
Source: Nat Commun. 2020 Apr 24;11:1998. doi: 10.1038/s41467-020-15855-z (PMC7181688; doi:10.1038/s41467-020-15855-z)
Supplement: Supplementary file 1 — Supplementary Information [file 41467_2020_15855_MOESM1_ESM.pdf]

Supplementary Information

Ethanol consumption inhibits T_{FH} cell responses and the development of autoimmune arthritis

Azizov et al.

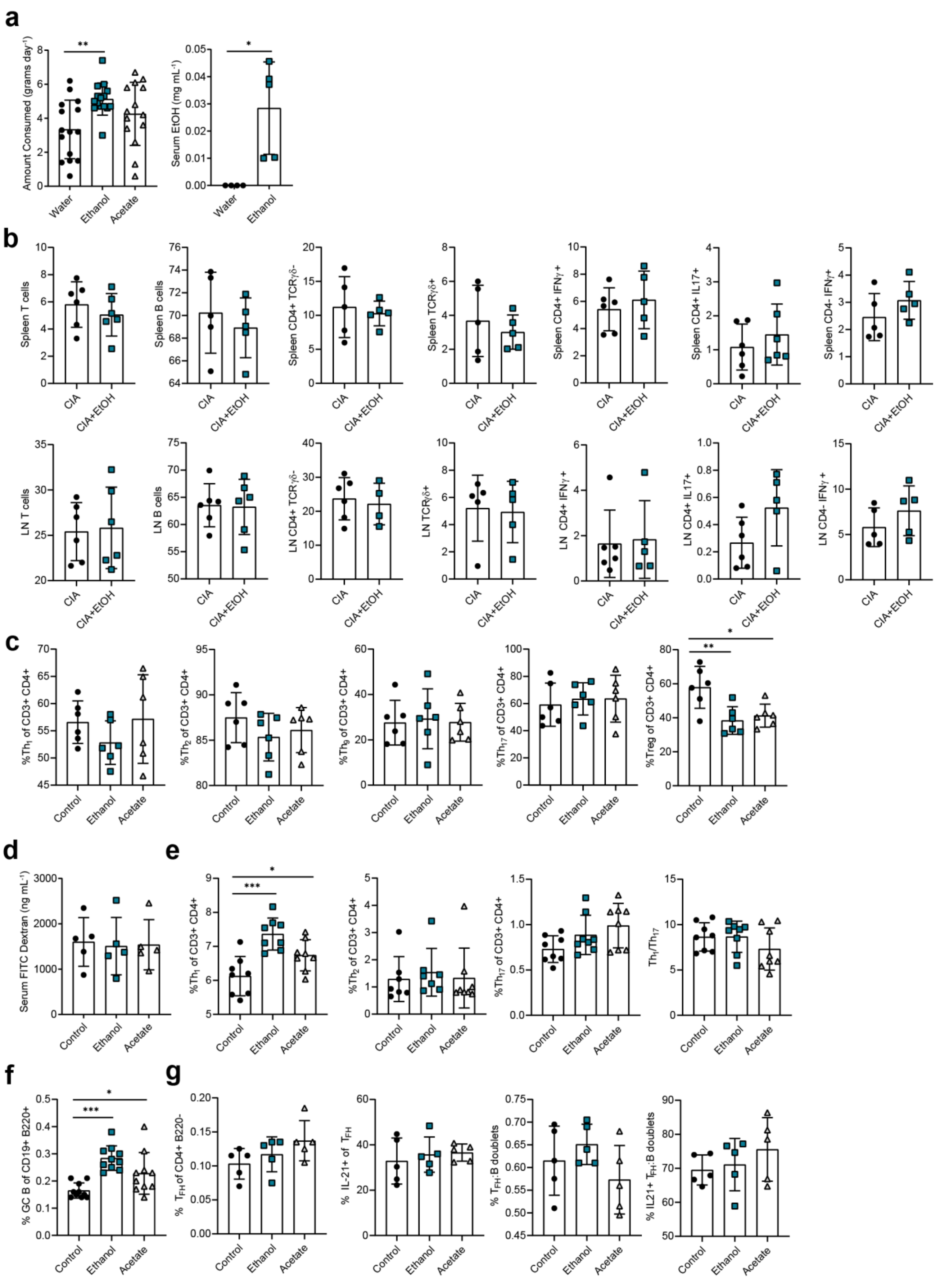

Supplementary Figure 1. The effect of ethanol and acetate on various immunological parameters. a Amount of water, ethanol, or acetate consumed in 24 hours (n = 5 mice per treatment), measured per mouse in grams per day in metabolic cages, and serum ethanol levels (in mg mL^{-1}) in mice fed with either water or ethanol (n = 5 mice per treatment per experiment). **b** Flow cytometry analysis of total T, B cells $CD4^{+}$

$TCR\gamma\delta^-$, $TCR\gamma\delta^+$, $CD4^+$ $IFN\gamma^+$, $CD4^+$ $IL17^+$, and $CD4^-$ $IFN\gamma^+$ in spleens and inguinal lymph nodes of CIA mice treated either with vehicle (CIA) or ethanol (CIA+EtOH) (n = 6 mice per condition per experiment). **c** In-vitro differentiation assay of naïve T cells to Th1, Th2, Th9, Th17, and T regulatory cells treated with either water (control), 10mM ethanol, or 0.5mM acetate (n=6 mice per experiment). **d** Gut leakiness of mice in steady state treated with either water (control, n=5 mice per experiment), ethanol (n=5 mice per experiment), or acetate (n=5 mice per experiment) measured with FITC-dextran gavage followed by serum FITC-dextran quantification. **e** Flow cytometry analysis of splenic Th1, Th2, Th17 cells and Th1/Th17 cell ratio in steady state mice treated with either water (control), ethanol or acetate (n=8 mice per condition per experiment). **f** Flow cytometry analysis of splenic GC B cells in steady state mice treated with either water (control), ethanol, or acetate (n=7 mice per condition per experiment). **g** Flow cytometry analysis of T_{FH} cells, IL-21 producing T_{FH} cell numbers, T_{FH}:B cell conjugates, as well as IL-21 producing T_{FH}:B cell conjugates in spleens of mice in steady state treated with either water (control), ethanol, or acetate (n=5 mice per condition per experiment). Data shown from one of three independent experiments and expressed as mean ± SD. Statistical difference was determined by one-way ANOVA (a (left graph), c, d, e, f, g) and two tailed Students t-test (a (right graph), b) p*= p<0.5; p**= p<0.01; p***= p<0.001.

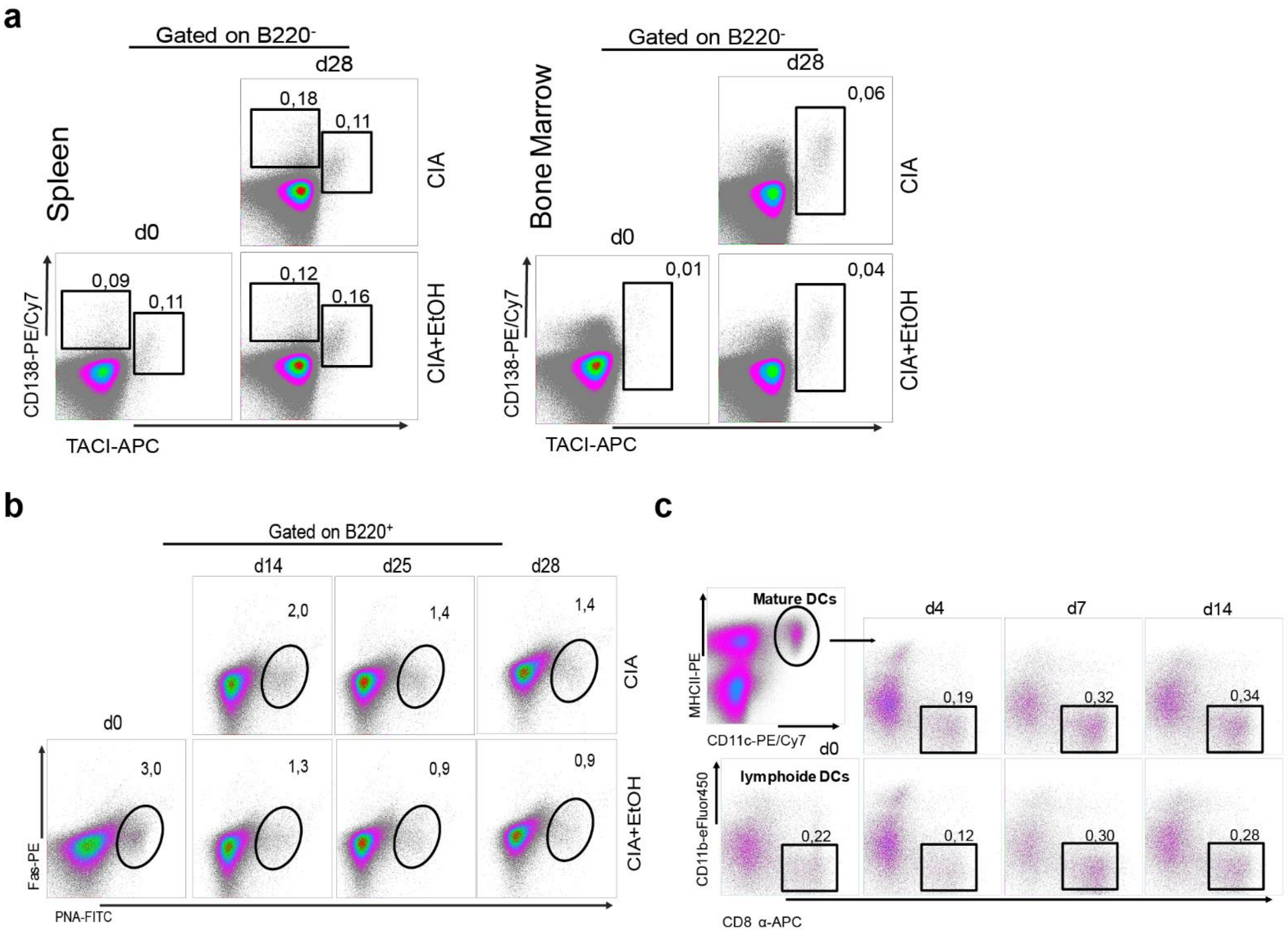

Supplementary Figure 2. Gating strategy of Plasmablasts, Plasma Cells, and DCs. a Flow cytometry gating strategy represented as dot plots of splenic and bone marrow plasmablasts (PB, $B220^{-}CD138^{+}TACI^{-}$) as well as plasma cells (PC, $B220^{-}$ $CD138^{+}$ $TACI^{+}$). **b** Flow cytometry gating strategy represented as dot plots where germinal center B cell populations are encircled and the average percentage noted above. **c** Flow cytometry gating strategy represented as dot plots determining lymphoid dendritic cells (DCs) in spleen. Shown are mature dendritic cells as gated on $CD11c^{+}$ $MHCII^{+}$ and further discriminated on the expression of $CD8\alpha^{+}$ $CD11b^{-}$ for lymphoid DCs (squared gates) at the indicated time points.

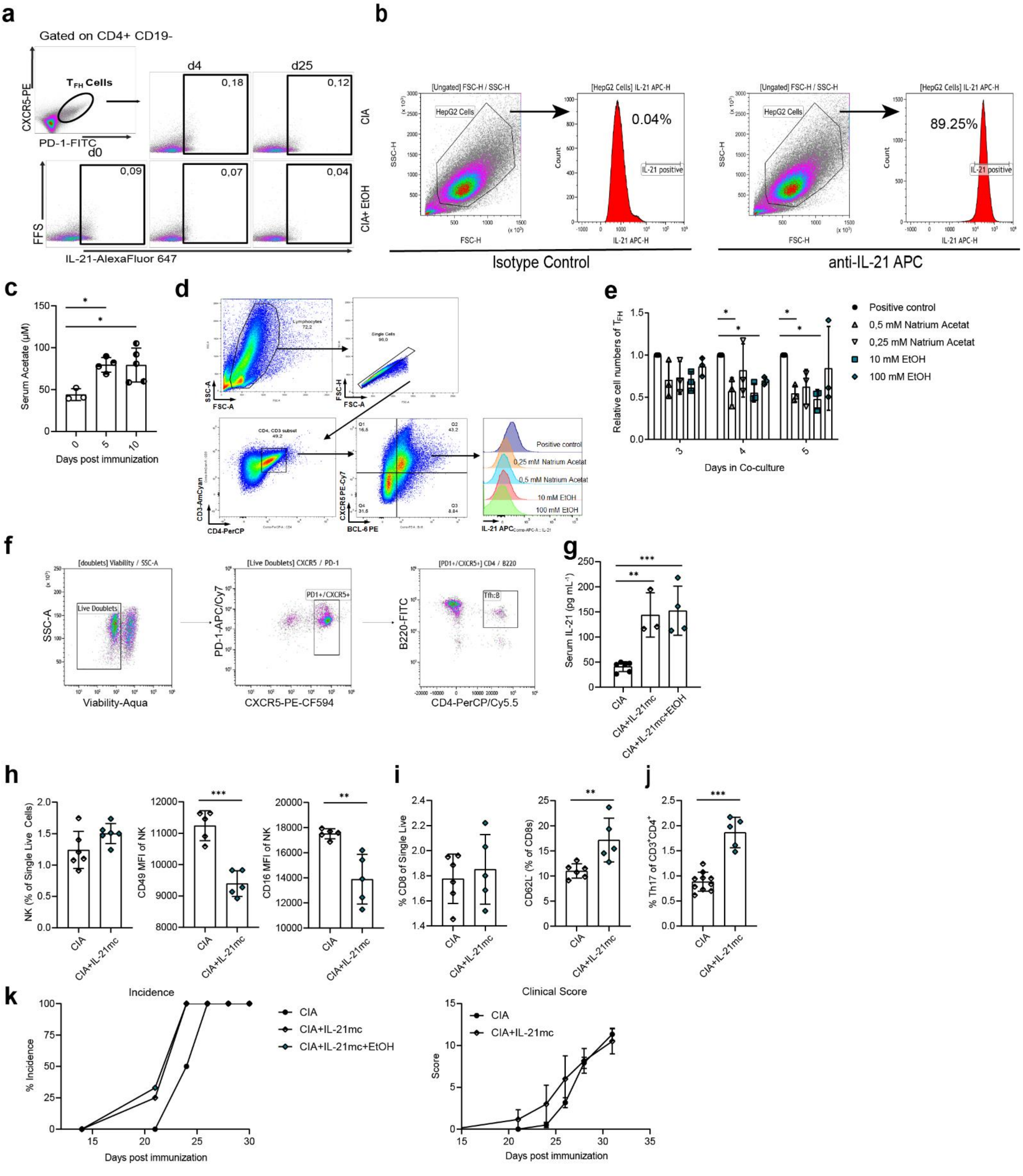

Supplementary Figure 3. T$_{FH}$ gating strategy and the effects of IL-21 overexpression on CIA. a Flow cytometry gating strategy of splenic T$_{FH}$ cells. **b** Confirmation of anti-IL-21 antibody by flow cytometry. **c** Serum acetate levels after the start of ethanol feeding and CIA induction (n = 5 mice per experiments). **d** Flow cytometry gating strategy of in-vitro differentiated T$_{FH}$ cells. **e** Relative cell numbers of live cells in in-vitro differentiation assay of T$_{FH}$ cells with no supplementation or with acetate (0,5 mM, 0,25 mM) or ethanol (100 mM, 10 mM) (n=1 mouse per condition per cell type per experiments). **f** Flow cytometry gating strategy of splenic T$_{FH}$:B cell conjugates. **g** Serum IL-21 levels quantified by ELISA in CIA mice either treated with vehicle (CIA, n = 6 mice per experiment) or IL-21 minicircle with (CIA+IL-21mc+EtOH, n = 4 mice per experiment) or without alcohol (CIA+IL-21mc, n = 3 mice per experiment). **h** Flow

cytometry analysis of Natural Killer cell numbers and expression levels (MFI) of activation markers CD49 and CD16 in CIA mice either treated with vehicle (CIA, n = 6 mice per experiment) or IL-21 minicircle (CIA+IL21mc, n = 6 mice per experiment). **i** Flow cytometry analysis of $CD8^+$ T cell numbers and their expression level (MFI) of CD62L in CIA mice either treated with vehicle (CIA, n = 6 mice per experiment) or IL-21 minicircle (CIA+IL21mc, n = 6 mice per experiment). **j** Flow cytometry analysis of Th17 cell numbers in the spleens of CIA mice either treated with vehicle (CIA, n = 10 mice per experiment) or IL-21 minicircle (CIA+IL21mc, n = 5 mice per experiment). **k** Incidence and clinical score of arthritis in CIA (n = 6 mice per experiment) mice treated with IL-21 minicircle DNA without (CIA+IL-21mc, n = 3 mice experiment) and with alcohol exposure mice (CIA+IL-21mc+EtOH, n = 4 mice per experiment). Data shown from one of three independent experiments and expressed as mean ± SD. Statistical difference was determined by one-way (c, g), two-way ANOVA (e, k) or Students t-test (h, i, j). p*= p<0.5; p**= p<0.01; p***= p<0.001.

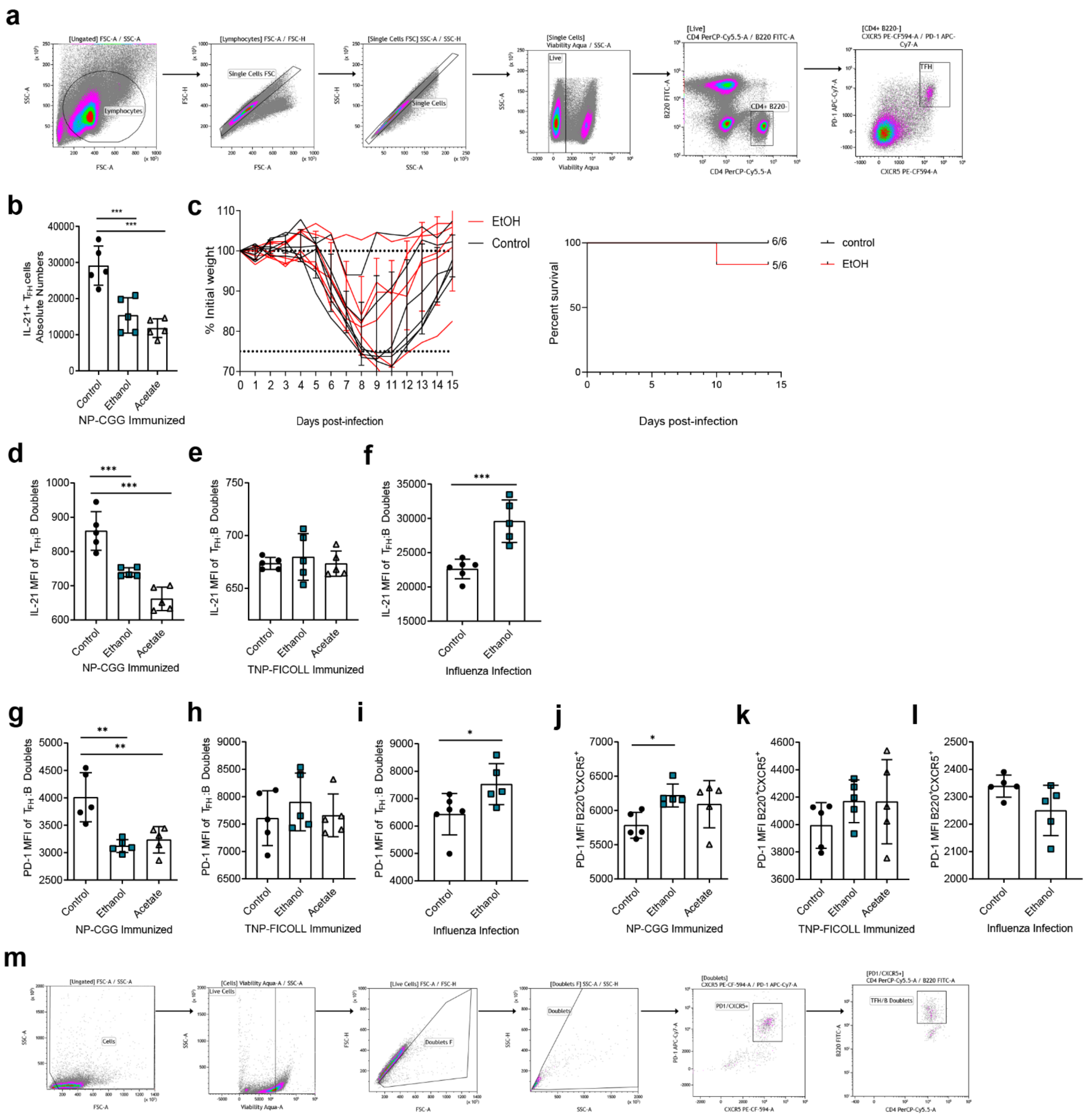

Supplementary Figure 4. T_{FH}:B conjugates reflect effects of alcohol and acetate consumption. a Flow cytometry gating strategy of splenic T_{FH} cells in NP-CGG, TNP-FICOLL immunizations and influenza infection model. **b** Absolute numbers of IL-21$^+$ T_{FH} cells analyzed by flow cytometry in the spleens of NP-CGG immunized mice treated with ethanol, and acetate (n = 5 mice per treatment, per experiment). **c** Weight loss and survival plot of influenza infection disease model depicted with loss/gain of weight (n = 6 mice per treatment per experiment). **d** Flow cytometry analysis of IL-21 MFI of T_{FH}:B cell conjugates in NP-CGG (n = 5 mice per experiment, per treatment), **e** in TNP-FICOLL-immunized (n = 5 mice per experiment, per treatment) mice treated with water (control), ethanol, or acetate **f** in influenza infected mice treated with water (control), or ethanol (n = 6 mice per treatment per experiment). **g** Flow cytometry analysis of PD-1 MFI of T_{FH}:B cell conjugates in NP-CGG (n = 5 mice per experiment, per treatment), **h** TNP-FICOLL-

immunized (n = 5 mice per experiment, per treatment), **i** influenza infected mice (n = 6 mice per treatment per experiment) treated with water (control), ethanol, or acetate. **j** Flow cytometry analysis of PD-1 MFI of splenic B cells (B220+ CXCR5+) in NP-CGG (n = 5 mice per experiment, per treatment), **k** TNP-FICOLL-immunized (n = 5 mice per experiment, per treatment), **l** influenza infected mice (n = 6 mice per treatment per experiment) treated with water (control), ethanol, or acetate. **m** Flow cytometry gating strategy of T_{FH}:B cell conjugates in in-vitro co-cultured sorted T_{FH} and B cells. Data shown from one of three independent experiments (Influenza infection model was independently repeated twice) and expressed as mean ± SD. Statistical difference was determined by one-way (b, d, e, g, h, j, k), two-way (c) ANOVA or Students t-test (f, i, l). p*= p<0.5; p**= p<0.01; p***= p<0.001.

Supplementary Table 1. Real-Time PCR primers reported as 5' to 3' sequence.

mβ-Actin	Fwd	TGTCCACCTTCCAGCAGATGT
	Rev	AGCTCAGTAACAGTCCGCCTAGA
mCCR3	Fwd	TCAACTTGGCAATTTCTGACCT
	Rev	GCATGGACGATAGCCAGG
mCXCL12	Fwd	TGCATCAGTGACGGTAAACCA
	Rev	TTCTTCAGCCGTGCAACAATC
mCXCR2	Fwd	ATGCCCTCTATTCTGCCAGAT
	Rev	GTGCTCCGGTTGTATAAGATGAC
mCXCR4	Fwd	GAAGTGGGGTCTGGAGACTAT
	Rev	TTGCCGACTATGCCAGTCAAG
mIL-1β	Fwd	CAGGCAGGCAGTATCACTCA
	Rev	AGGTGCTCATGTCCTCATCC
mIL-4	Fwd	GGTCTCAACCCCCAGCTAGT
	Rev	GCCGATGATCTCTCTCAAGTGAT
mIL-6	Fwd	TCCTTCCTACCCCAATTTCC
	Rev	GCCACTCCTTCTGTGACTCC
mIL-9	Fwd	ATGTTGGTGACATACATCCTTGC
	Rev	TGACGGTGGATCATCCTTCAG
mIL-13	Fwd	CCTGGCTCTTGCTTGCCTT
	Rev	GGTCTTGTGTGATGTTGCTCA
mIL-33	Fwd	CGGGTACCAAGCATGAAG
	Rev	TCAACAGACGCAGCAAATG
ICOS	Fwd	CTGTAAAGCACACAGGCAGC
	Rev	TGGCCTGCTATACTCATTAGGG
IL-21R	Fwd	GGCTGCCTTACTCCTGCTG
	Rev	TCATCTTGCCAGGTGAGACTG
